# Improved phylogenetic resolution within Siphonophora (Cnidaria) with implications for trait evolution

**DOI:** 10.1016/j.ympev.2018.06.030

**Published:** 2018-06-22

**Authors:** Catriona Munro, Stefan Siebert, Felipe Zapata, Mark Howison, Alejandro Damian-Serrano, Samuel H. Church, Freya E. Goetz, Philip R. Pugh, Steven H.D. Haddock, Casey W. Dunn

**Affiliations:** a Department of Ecology and Evolutionary Biology, Brown University, Providence, RI 02912, USA; b Department of Molecular & Cellular Biology, University of California Davis, Davis, CA 95616, USA^2^; c Department of Ecology and Evolutionary Biology, University of California Los Angeles, Los Angeles, CA 90095, USA; d Brown Data Science Practice, Brown University, Providence, RI 02912, USA; e Watson Institute for International and Public Affairs, Brown University, Providence, RI 02912, USA^2^; f Department of Organismic and Evolutionary Biology, Harvard University, Cambridge, MA 02138, USA^2^; g Smithsonian Institution, National Museum of Natural History, Washington, DC 20560, USA^2^; h National Oceanography Centre, Southampton SO14 3ZH, UK; i Monterey Bay Aquarium Research Institute, Moss Landing, CA 95039, USA; j Department of Ecology and Evolutionary Biology, Yale University, New Haven, CT 06520, USA

## Abstract

Siphonophores are a diverse group of hydrozoans (Cnidaria) that are found at most depths of the ocean - from the surface, like the familiar Portuguese man of war, to the deep sea. They play important roles in ocean ecosystems, and are among the most abundant gelatinous predators. A previous phylogenetic study based on two ribosomal RNA genes provided insight into the internal relationships between major siphonophore groups. There was, however, little support for many deep relationships within the clade Codonophora. Here, we present a new siphonophore phylogeny based on new transcriptome data from 29 siphonophore species analyzed in combination with 14 publicly available genomic and transcriptomic datasets. We use this new phylogeny to reconstruct several traits that are central to siphonophore biology, including sexual system (monoecy vs. dioecy), gain and loss of zooid types, life history traits, and habitat. The phylogenetic relationships in this study are largely consistent with the previous phylogeny, but we find strong support for new clades within Codonophora that were previously unresolved. These results have important implications for trait evolution within Siphonophora, including favoring the hypothesis that monoecy arose at least twice.

## 1. Introduction

**Fig. 1:**
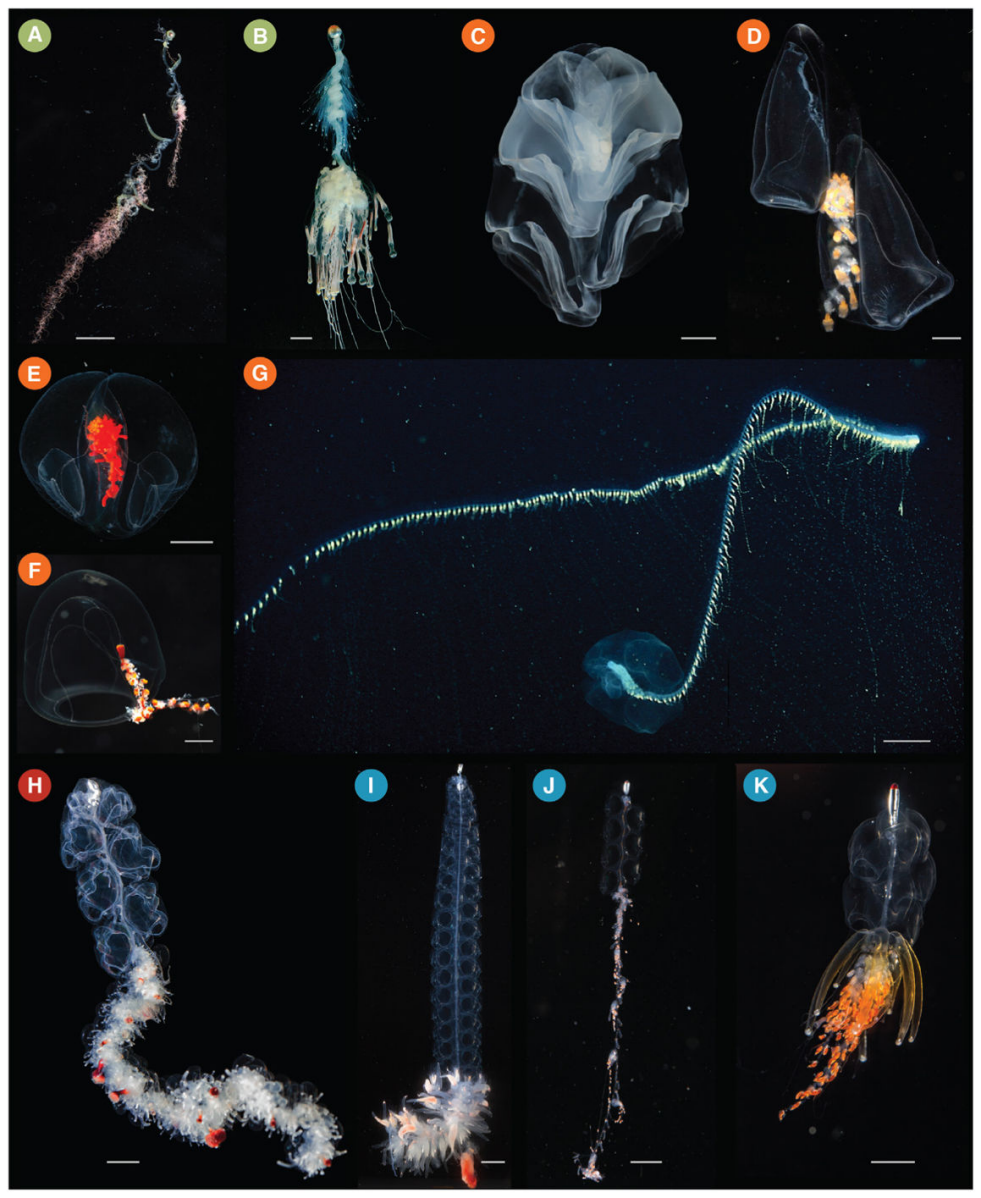
Photographs of living siphonophores. Colored circles correspond to the clades shown in Fig. 3 as follows: Cystonectae (A and B), Calycophorae (C–G), Apolemiidae (H), and Clade A within Euphysonectae (I–K). (A) *Rhizophysa eysenhardtii*, scale bar = 1 cm. (B) *Bathyphysa conifera*, scale bar = 2 cm. (C) *Hippopodius hippopus*, scale bar = 5 mm. (D) *Kephyes hiulcus*, scale bar = 2 mm. (E) *Desmophyes haematogaster*, scale bar = 5 mm. (F) *Sphaeronectes christiansonae*, scale bar = 2 mm. (G) *Praya dubia*, scale bar = 4 cm. (H) *Apolemia* sp., scale bar = 1 cm. (I) *Lychnagalma utricularia*, scale bar = 1 cm. (J) *Nanomia* sp., scale bar = 1 cm. (K) *Physophora hydrostatica*, scale bar = 5 mm. Photo credits: S. Siebert (C,H,I,K), S. Haddock (A,D,E,F), R. Sherlock (B), MBARI (G), C. Munro (J). (For interpretation of the references to colour in this figure legend, the reader is referred to the web version of this article.)

**Fig. 2:**
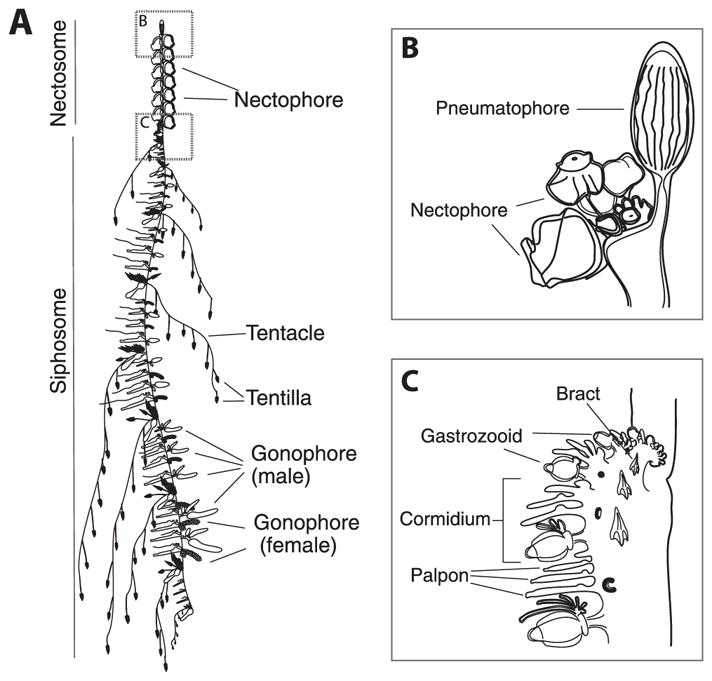
Schematic of the siphonophore *Nanomia bijuga*, oriented with the anterior of the colony at the top of the page, and the ventral side to the left. Adapted from http://commons.wikimedia.org/wiki/File:Nanomia_bijuga_whole_animal_and_growth_zones.svg, drawn by Freya Goetz. (A) Overview of the whole mature colony. (B) Inset of the pneumatophore and nectosomal growth zone. A series of buds give rise to nectophores. (C) Inset of the siphosomal growth zone. Probuds subdivide to give rise to zooids in repeating units (cormidia). The gastrozooid (specialized feeding polyp) is the posterior-most zooid within each cormidium.

Siphonophores (Figs. 1 and 2) are among the most abundant gelatinous predators in the open ocean, and have a large impact on ocean ecosystems (Choy et al., 2017; Pagès et al., 2001; Pugh, 1984; Pugh et al., 1997; Purcell, 1981; Williams and Conway, 1981). Siphonophores, which belong to Hydrozoa (Cnidaria), are found at most depths in the ocean, with the deepest recorded species found around 4,300 m (Lindsay, 2005). The most familiar species is the Portuguese man of war *Physalia physalis*, which floats at the surface and can wash up conspicuously onto beaches (Totton, 1960). Most species are planktonic, living in the water column, where some grow to be more than 30 m in length (Mackie et al., 1987). There is also a small clade of benthic siphonophores, Rhodaliidae, that are tethered to the bottom for part of their lives (Pugh, 1983). There are currently 187 valid described siphonophore species (Schuchert, 2018).

Siphonophores remain poorly known, in large part because they are fragile and difficult to collect. They have, however, been of great interest for more than 150 years due to their unique structure and development (Mackie et al., 1987; Mapstone, 2014). Like many other cnidarians, they are colonial: they grow by incomplete asexual reproduction. Each colony arises from a single embryo that forms the protozooid, the first body. One or two growth zones (Fig. 2) then arise and asexually produce other genetically identical zooids that remain attached (Carré, 1967, 1969; Carré and Carré, 1991, 1993). In some species additional zooids are added outside the growth zone along the siphosomal stem (Siebert et al., 2013). The zooids are each homologous to a solitary animal, but are physiologically integrated (Dunn and Wagner, 2006; Mackie et al., 1987; Totton, 1965). Siphonophores differ significantly from other colonial animals in their colony structure and development – their zooids are highly functionally specialized and arranged in precise, repeating, species-specific patterns (Beklemishev, 1969; Cartwright and Nawrocki, 2010). Zooids are specialized for a range of functions, including feeding, reproduction, or swimming (Fig. 2) (Dunn and Wagner, 2006).

Understanding the unique ecology, morphology, and development of siphonophores requires a well-resolved phylogeny of the group. The relationship of siphonophores to other hydrozoans has been difficult to determine (Cartwright et al., 2008; Cartwright and Nawrocki, 2010; Kayal et al., 2013, 2015, 2018; Zapata et al., 2015), but there has been progress on their internal relationships. A phylogeny (Dunn et al., 2005) based on two genes (16S, 18S) from 52 siphonophore taxa addressed several long standing questions about siphonophore biology, including the relationships of the three historically recognized groups, Cystonectae, Physonectae, and Calycophorae. Cystonectae was found to be sister to all other siphonophores, while Calycophorae were nested within “Physonectae”. The name Codonophora was given to this clade of “Physonectae” and Calycophorae (Dunn et al., 2005).

Major questions remained after this early work, though. In particular, there was little support for important deep relationships within Codonophora. Understanding these relationships is key to resolving the evolution of several traits of importance, including sexual systems (monoecy versus dioecy) and the gain and loss of particular zooids, such as palpons (Fig. 2). Here we present a broadly sampled phylogenetic analysis of Siphonophora that considers transcriptomic data from 33 siphonophore species and 10 outgroup species (2 outgroups were subsequently excluded due to poor sampling). Using 1,423 genes, we find strong support for many relationships found in the earlier phylogeny (Dunn et al., 2005), and also provide new resolution for key relationships that were unresolved in that previous study. Using this phylogeny, we reconstruct the evolutionary history of characters central to the unique biology of siphonophores, including zooid type, life history traits, and vertical habitat use.

## 2. Material and methods

All scripts for the analyses are available in a git repository at https://github.com/caseywdunn/siphonophore_phylogeny_2017. The most recent commit at the time of the analysis presented here was 1501118c with tag “paper_v2”.

### 2.1. Collecting

Specimens were collected in the north-eastern Pacific Ocean, Mediterranean, and the Gulf of California (see Table 1). Collection data on all examined specimens, a description of the tissue that was sampled from the colony, collection mode, sample processing details, mRNA extraction methods, sequencing library preparation methods, and sequencing details are summarized in the file Supplementary data 1 (also found in the git repository). Monterey Bay and Gulf of California specimens were collected by remotely operated underwater vehicle (ROV) or during blue-water SCUBA dives. *Chelophyes appendiculata* and *Hippopodius hippopus* (Fig. 1C) specimens were collected in the bay of Villefranche-sur-Mer, France, during a plankton trawl on 13 April 2011. Available physical vouchers have been deposited at the Museum of Comparative Zoology (Harvard University), Cambridge, MA, the Pea-body Museum of Natural History (Yale University), New Haven, CT, or had been previously deposited at the Smithsonian National Museum of Natural History, Washington, DC. Accession numbers are given in Supplementary data 1. In cases where physical vouchers were unavailable we provide photographs to document species identity (see git repository: https://github.com/caseywdunn/siphonophore_phylogeny_2017/tree/master/supplementary_info/photographic_vouchers).

### 2.2. Sequencing

When possible, specimens were starved overnight in filtered sea-water at temperatures close to ambient water temperatures at the time of specimen collection. Subsequently, mRNA was extracted directly from tissue using a variety of methods (Supplementary data 1): Magnetic mRNA Isolation Kit (NEB, #S1550S), Invitrogen Dynabeads mRNA Direct Kit (Ambion, #61011), Zymo Quick RNA MicroPrep (Zymo #R1050), or from total RNA after Trizol (Ambion, #15596026) extraction and through purification using Dynabeads mRNA Purification Kit (Ambion, #61006). In case of small total RNA quantities, only a single round of bead purification was performed. Extractions were performed according to the manufacturer’s instruction. All samples were DNase treated (TURBO DNA-free, Invitrogen #AM1907; or on column DNase treatment with Zymo Quick RNA MicroPrep). Libraries were prepared for sequencing using the Illumina TruSeq RNA Sample Prep Kit (Illumina, #FC-122-1001, #FC-122-1002), the Illumina TruSeq Stranded Library Prep Kit (Illumina, #RS-122-2101) or the NEBNext RNA Sample Prep Master Mix Set (NEB, #E6110S). We collected long read paired end Illumina data for *de novo* transcriptome assembly. In the case of large tissue inputs, libraries were sequenced separately for each tissue, subsequently subsampled and pooled *in silico*. Libraries were sequenced on the HiSeq 2000, 2500, and 3000 sequencing platforms. Summary statistics for each library are given in the file Supplementary data 2. All sequence data have been deposited in the NCBI sequence read archive (SRA) with Bioproject accession number PRJNA255132.

### 2.3. Analysis

New data were analysed in conjunction with 14 publicly available datasets (Chapman et al., 2010; Dunn et al., 2013; Fidler et al., 2014; Lehnert et al., 2012; Philippe et al., 2009; Putnam et al., 2007; Sanders and Cartwright, 2015; Sanders et al., 2014; Zapata et al., 2015), with a total number of 43 species. Sequence assembly, annotation, homology evaluation, gene tree construction, parsing of genes trees to isolate orthologous sequences, and supermatrix construction were conducted with Agalma (commit 6bd9988, running BioLite commit 784edc6) (Dunn et al., 2013; Guang et al., 2017). This workflow integrates a variety of existing tools (Altschul et al., 1990; Enright et al., 2002; Grabherr et al., 2011; Katoh and Standley, 2013; Langmead and Salzberg, 2012; Li and Dewey, 2011; Li et al., 2009; Sukumaran and Holder, 2010; Talavera and Castresana, 2007) and new methods.

Two outgroup species, *Atolla vanhoeffeni* and *Aegina citrea*, were removed from the final supermatrix due to low gene occupancy (gene sampling of 17.0% and 17.3% respectively in a 60% occupancy matrix with 3379 genes). The final analyses presented here consider 33 siphonophore species and 8 outgroup species. This includes new data for 30 species. In the final analyses, we sampled 1,423 genes to generate a supermatrix with 80% occupancy and a length of 395,699 amino acids (Fig. S1).

We used ModelFinder (Kalyaanamoorthy et al., 2017), as implemented in IQTree v1.6.3 (Nguyen et al., 2015), to assess relative model fit. ModelFinder selected JTT + Empirically counted frequencies from alignment + FreeRate model with 7 categories based on the Bayesian Information Criterion. To assess the robustness of our results, we conducted phylogenetic analyses using multiple software programs, methods (Maximum likelihood (ML) and Bayesian Inference (BI)), and models (including the model selected by ModelFinder and several other commonly used models). Maximum likelihood (ML) analyses were conducted with RAxML v8.2.0 (Stamatakis, 2006) and IQTree v1.6.3 (Hoang et al., 2018; Nguyen et al., 2015). Bayesian Inference (BI) were conducted with Phylobayes v. 1.7a-mpi (Lartillot et al., 2009). Sequence alignments, sampled and consensus trees, and voucher information are available in the git repository. Tree figures were rendered with ggtree (Yu et al., 2016).

**Fig. 3:**
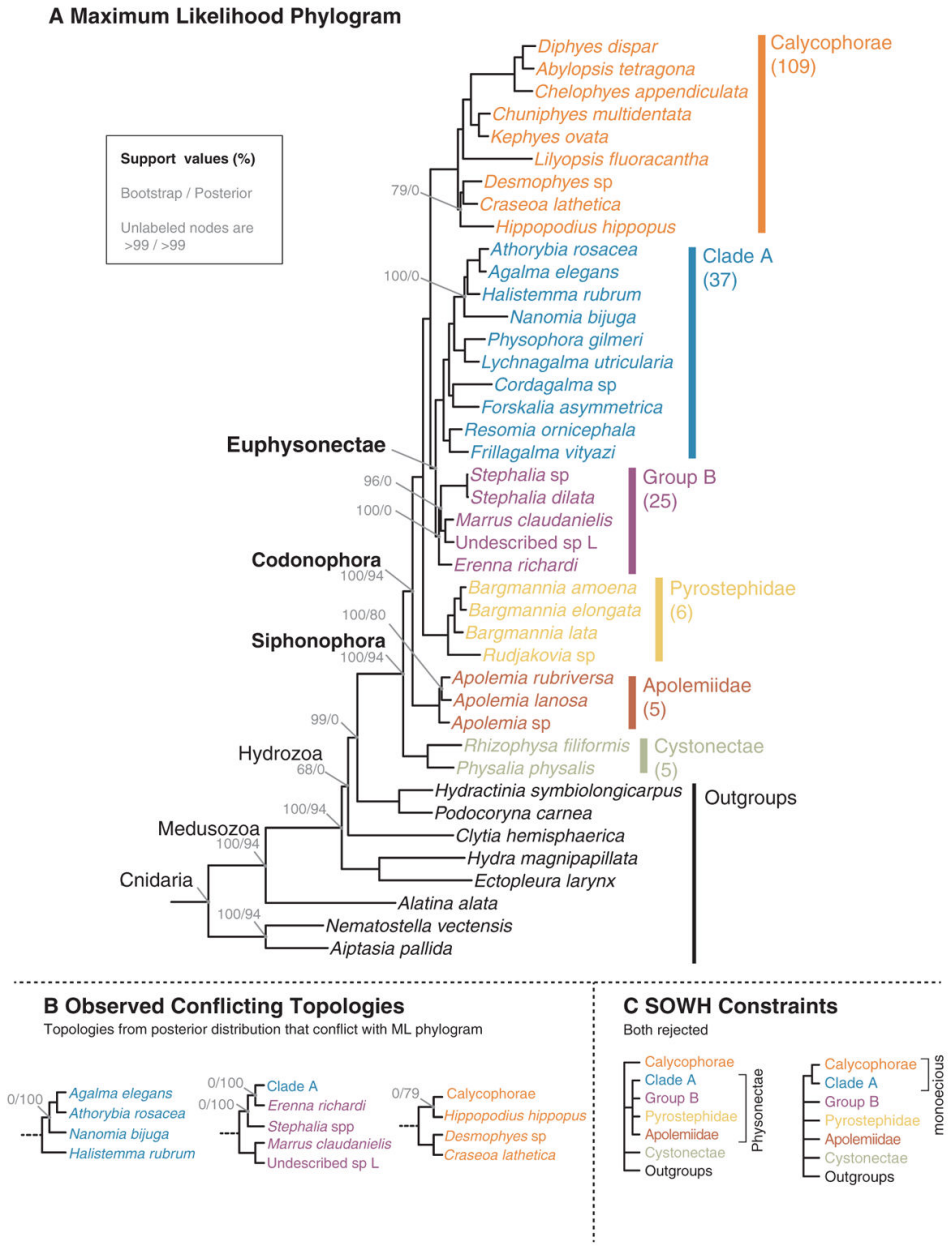
(A) Maximum likelihood (ML) phylogram with bipartition frequencies from the ML bootstraps and the Bayesian posterior distribution of trees. Unlabeled nodes have support > 0.99 for both bootstraps and posteriors. The numbers of valid described species estimated to be based in each clade based on taxonomy are shown below each clade name on the right. (B) The topologies found in the posterior distribution of trees that conflict with the ML tree. (C) The topologies evaluated by the SOWH tests. For more details on the SOWH topologies refer to Fig. S2.

RAxML ML analyses were conducted on the unpartitioned super-matrix using the WAG+Γ model of amino acid substitution (Fig. 3A). RAxML bootstrap values were estimated using 1000 replicates. IQTree ML analyses were run under JTT + Empirically counted frequencies from alignment + FreeRate model with 7 categories, the best model identified by ModelFinder, and the commonly used models GTR + Optimized base frequencies + Free rate model with 6 categories and WAG + Optimized base frequencies + Free rate model with 6 categories (Fig. S10).

BI was conducted in phylobayes using two different models: fixed-state WAG+Γ (Fig. S12) and CAT-Poisson (Fig. S11). Eight chains were run under the CAT-Poisson model. Four chains were run under WAG+Γ, these runs did not converge (maxdiff = 1, mean-diff = 0.0130273). The CAT-Poisson runs did not converge (max-diff = 1, meandiff = 0.0565898). Closer inspection revealed that chain 1 and chain 3 were stuck in local maxima with low likelihood relative to other chains after 1,405 and 4,695 iterations. These two chains were excluded from the analyses, and the results presented here are based on the remaining 6 CAT-Poisson chains (maxdiff = 1, mean-diff = 0.0185032). Visual inspection of the traces indicated that a burn in of 400 trees was sufficient for all CAT-Poisson runs. This left 17,893 trees in the CAT-Poisson posterior.

We used the Swofford-Olsen-Waddell-Hillis (SOWH) test (Swofford et al., 1996) to evaluate two hypotheses (Fig. 3C, S2): (i) “Physonectae” is monophyletic (Totton, 1965); (ii) monoecious species are monophyletic (Dunn et al., 2005). The sexual mode of *Rudjakovia* is undescribed, but preliminary observations suggest that they are monoecious, so we include *Rudjakovia* as a monoecious species in this test. We used SOWHAT (Church et al., 2015a) dev. version 0.39 (commit fd68ef57) to carry out the SOWH tests in parallel, using the default options and an initial sample size of 100 (analysis code can be found in the git repository). For each hypothesis we defined a topology with a single constrained node that was inconsistent with the most likely topology (Fig. 3). We used a threshold for significance of 0.05 and following the initial 100 samples, we evaluated the confidence interval around the p-value to determine if more samples were necessary.

Morphological character data used in trait mapping were obtained from the literature or direct observation of available voucher material. Depth distribution data were queried from the MBARI VARS database (http://www.mbari.org/products/research-software/video-annotation-and-reference-system-vars/) (Schlining and Stout, 2006). We used stochastic character mapping to infer the most probable evolution of traits on the tree in R using the phytools package (Huelsenbeck et al., 2003; Revell, 2012). For continuous character traits, model fit was tested using fitContinuous in the geiger R package. Subsequent analyses were conducted in R and integrated into this manuscript with the knitr package. See Supplementary Information for R package version numbers.

## 3. Results and discussion

### 3.1. Species phylogeny and hypothesis testing

The phylogenetic relationships recovered in this study received strong support across analysis methods (Fig. 3A), with a couple of localized exceptions (Fig. 3B and S11). All of the ML analyses were congruent with each other, regardless of model and software used (Fig. S10). These ML results were also congruent with the Phylobayes BI WAG+Γ analyses (Fig. S12). The Phylobayes BI CAT-Poisson result (Fig. S11), however, had a strongly supported topology that differed (Fig. 3B) from the ML topology in localized regions as described below. The fact that the Phylobayes BI WAG+Γ is consistent with the WAG (and other) ML analyses suggests that the different topology recovered in the Phylobayes BI CAT-Poisson analyses is due to the different model rather than different software or methods. Here we take the conservative approach of considering relationships that differ between the Phylobayes BI CAT-Poisson analyses and other analyses to be unresolved.

Most clades are consistent with those found in a previous study based on two genes (16S and 18S ribosomal RNA) (Dunn et al., 2005). Relationships that receive strong support in both include the placement of Cystonectae as sister to Codonophora (the clade that includes all other siphonophores), the placement of Apolemiidae as sister to all other codonophorans, and the placement of Calycophorae within the paraphyletic “Physonectae”. Multiple nodes that were not resolved in the previous two-gene analysis receive strong support in the present 1,423-gene transcriptome analyses. There is strong support for Pyrostephidae as sister to all other non-apolemiid codonophorans. We provisionally refer here to Pyrostephidae as the clade including *Rudjakovia* sp., although sampling of *Pyrostephos vanhoeffeni* is needed in order to determine if *Rudjakovia* sp. falls within Pyrostephidae or is sister to it. Within the clade that is sister to Pyrostephidae, we find two main clades, Calycophorae and a clade we here name Euphysonectae (Fig. 3A). It includes the remaining non-apolemiid, non-pyrostephid “Physonectae”. We define Euphysonectae as the clade consisting of *Agalma elegans* and all taxa that are more closely related to it than to

#### Diphyes dispar

In ML analyses and BI WAG analyses, Euphysonectae consists of two reciprocally monophyletic groups that we here provisionally refer to as Clade A and Group B (Fig. 3A). In BI CAT-Poisson analyses, Group B is paraphyletic (Fig. 3B). The presence of an involucrum, a fold around the base of the cnidoband (Totton, 1965), is a potential synapomorphy for Clade A. Species of Clade A also have a descending mantle canal within the nectophores (Figs. S6 and S18), a structure that is also present in some calycophorans. Members of Clade A are also monoecious (Fig. 5). There is not a clear synapomorphy for Group B. Within Group B there is high support for the placement of *Erenna richardi* in ML analyses and BI WAG (Figs. 3 and S12), but it is placed as sister to Clade A in BI CAT-Poisson analyses (Fig. 3B). More taxon sampling will be required to determine the relationship of species within this group.

Within Clade A, *Physophora gilmeri* along with *Lychnagalma utricularia* (Fig. 1I) (both not included in the previous phylogeny) are sister to Agalmatidae, a clade restricted to *Agalma*, *Athorybia*, *Melophysa*, *Halistemma* and *Nanomia* (Dunn et al., 2005; Pugh, 2006). In the rDNA study, *P. hydrostatica* (the presumed sister species to *P. gilmeri*) was sister to Forskaliidae with low support. The position of *Cordagalma cordiforme* (=*C. ordinatum*) (Pugh, 2016) was previously unresolved, while in this analysis *Cordagalma* sp. is in a clade with *Forskalia asymmetrica*, falling outside of Agalmatidae. Placement of *Cordagalma* outside Agalmatidae is consistent with previous analyses of molecular and morphological data (Dunn et al., 2005; Pugh, 2006).

Within Calycophorae, taxon sampling is less comprehensive here than in the previous study. The caly cophoran relationships that can be investigated, however, are in broad agreement with the previous analysis. Calycophorans have in the past been split into two groups, prayomorphs and diphyomorphs, based on morphology after Mackie et al. (1987). As in the previous study, the results presented here indicate that the prayomorphs are paraphyletic with respect to the di-phyomorphs. In the previous study, the relationship between *C. lathetica* and the clade including *H. hippopus* was unresolved. In this study, *Craseoa lathetica* and *Desmophyes* sp. are sister to *Hippopodius hippopus* in ML and BI-WAG analyses with high support, while in BI CAT-Poisson analyses, *H. hippopus* is sister to *Lilyopsis fluoracantha* and the diphyomorphs (Figs. 3B and S11).

Using the Swofford-Olsen-Waddell-Hillis (SOWH) test (Swofford et al., 1996), we evaluated the following two alternative phylogenetic hypotheses against the most likely tree topology (Fig. 3C): (i) monophyletic Physonectae, (ii) monophyletic monoecious siphonophores. In both tests the alternative hypothesis was rejected (p-value < 0.01, confidence interval: < 0.001–0.03, Fig. S2).

**Fig. 4:**
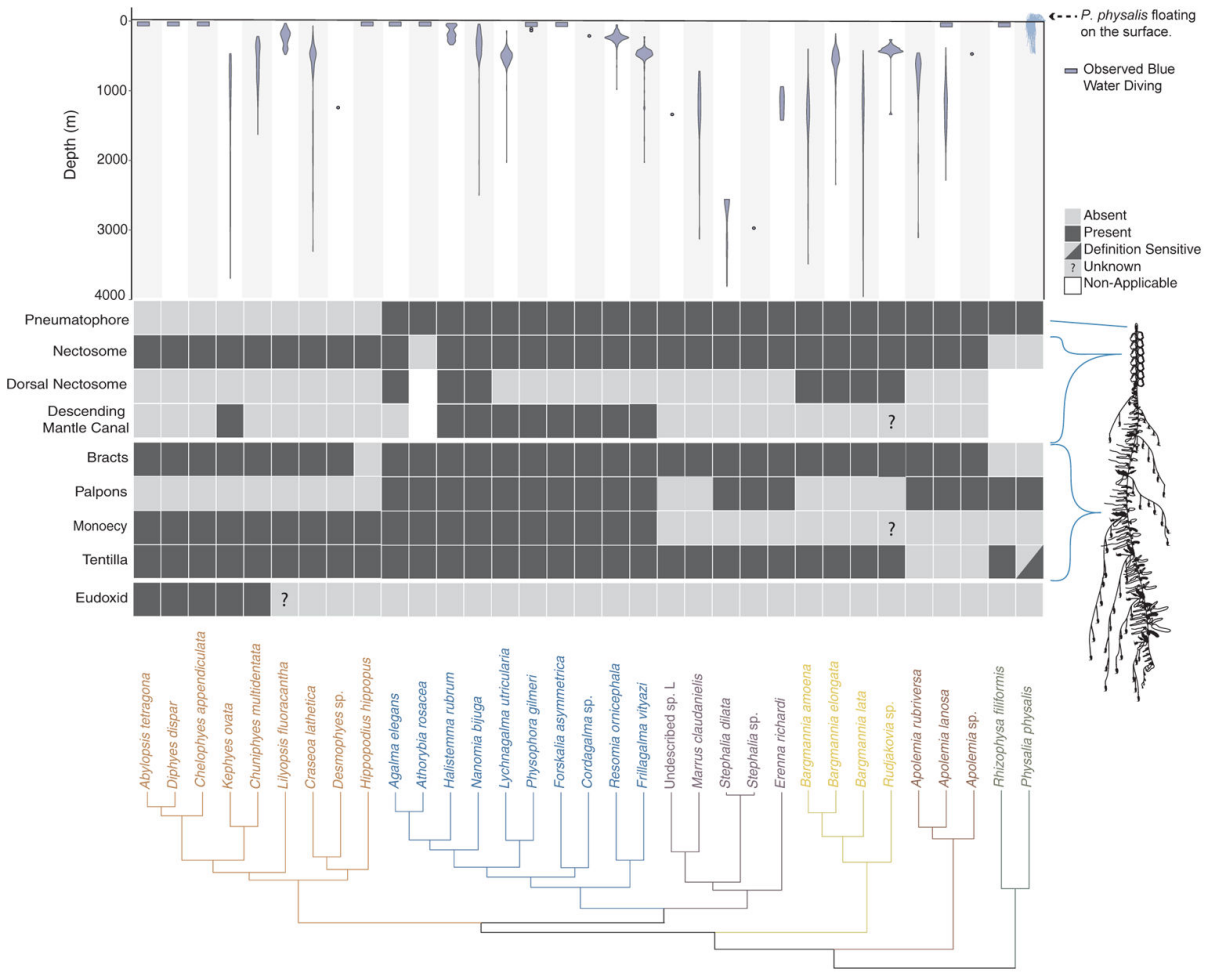
Siphonophore phylogeny showing the distribution of the main anatomical characters and the bathymetric distributions of the different species. Bottom: siphonophore phylogeny, colored by clade. Middle: diagram showing the presence/absence of traits across Siphonophora, with the physical location of the trait shown on a schematic of *Nanomia bijuga* (schematic by Freya Goetz). Top: Bathymetric distribution of siphonophore species. *Physalia* illustration by Noah Schlottman, taken from http://phylopic.org/. (For interpretation of the references to colour in this figure legend, the reader is referred to the web version of this article.)

The broad taxon sampling and more extensive gene sampling of this phylogeny provide new evidence for the relationships between major siphonophore clades within Codonophora, specifically between Pyrostephidae, Calycophorae, and the newly named Euphysonectae. This opens up new questions about key relationships within both Calycophorae and Euphysonectae – where future transcriptome sampling efforts should be focused. Within Euphysonectae, two clades (Clade A and Group B) are hypothesized, although there is weaker support for Group B (Fig. 3A and B). Expanding sampling of species that probably fall in Group B, including other *Erenna* species, rhodaliids, and relatives of Undescribed sp L, will greatly expand our understanding of these two groups and perhaps provide evidence of Group B synapomorphies. Similarly, within Calycophorae, increased taxon sampling is needed. This study, and the previous phylogenetic study (Dunn et al., 2005), suggest that the prayomorphs are paraphyletic, but for slightly different reasons given the different sampling of the analyses. In Dunn et al. (2005), a clade of prayomorphs including *Praya dubia* (Fig. 1G), *Nectadamas diomedeae*, and *Nectopyramis natans* (not included in this study) were found to be sister to all other calycophorans, while in this study, the prayomorph *Lilyopsis fluoracantha* (not included in the previous study) is found in a clade including diphyomorph calycophorans that is sister to all other prayomorphs. Expanded transcriptome sequencing, particularly *P. dubia* or a nectopyramid, but also extensive sampling across the major prayomorph and diphyomorph groups, will expand our understanding of relationships within Calycophorae. This will be especially important for understanding trait evolution within Calycophorae, for example, the release of eudoxids (Fig. 4), or the arrangement of male and female zooids along the stem (see Section 3.2 below).

### 3.2. Evolution of monoecy

In all siphonophores, each gonophore (sexual medusa that produces gametes) is either male or female. Within each siphonophore species, colonies are either monoecious (male and female gonophores are on the same colony) or dioecious (male and female gonophores are on different colonies). Previous analysis suggested that the common ancestor of siphonophores was dioecious, and was consistent with a single gain of monoecy within Codonophora and no secondary losses (Dunn et al., 2005). The better-resolved tree in the current analyses indicates that the evolution of monoecy is more complicated than this. The two clades of monoecious siphonophores, Calycophorae and Clade A (Fig. 3A), do not form a monophyletic group. This is because Group B, which contains dioecious species, is also descended from their most recent common ancestor. The SOWH test strongly rejects the placement of the monecious clades Calycophorae and Clade A as a group that excludes Group B (Figs. 3C and S2). The positions of the only two taxa from Group B that were included in the previous analysis (Dunn et al., 2005), *Erenna* and *Stephalia*, were unresolved in that study. This difference in conclusions regarding trait evolution, therefore, does not reflect a contradiction between alternative strongly supported results, but the resolution of earlier polytomies in a way that indicates there has been homoplasy in the evolution of monoecy.

The distribution of monoecy is consistent with two potential scenarios (Fig. 4). In the first, there is a single shift from dioecy to monoecy along the branch that gave rise to the most recent common ancestor of Calycophorae and Euphysonectae, followed by a shift back to dioecy along the branch that gave rise to Group B. In the second, monoecy arose twice: once along the branch that gave rise to Clade A and again along the branch that gave rise to Calycophorae.

**Fig. 5:**
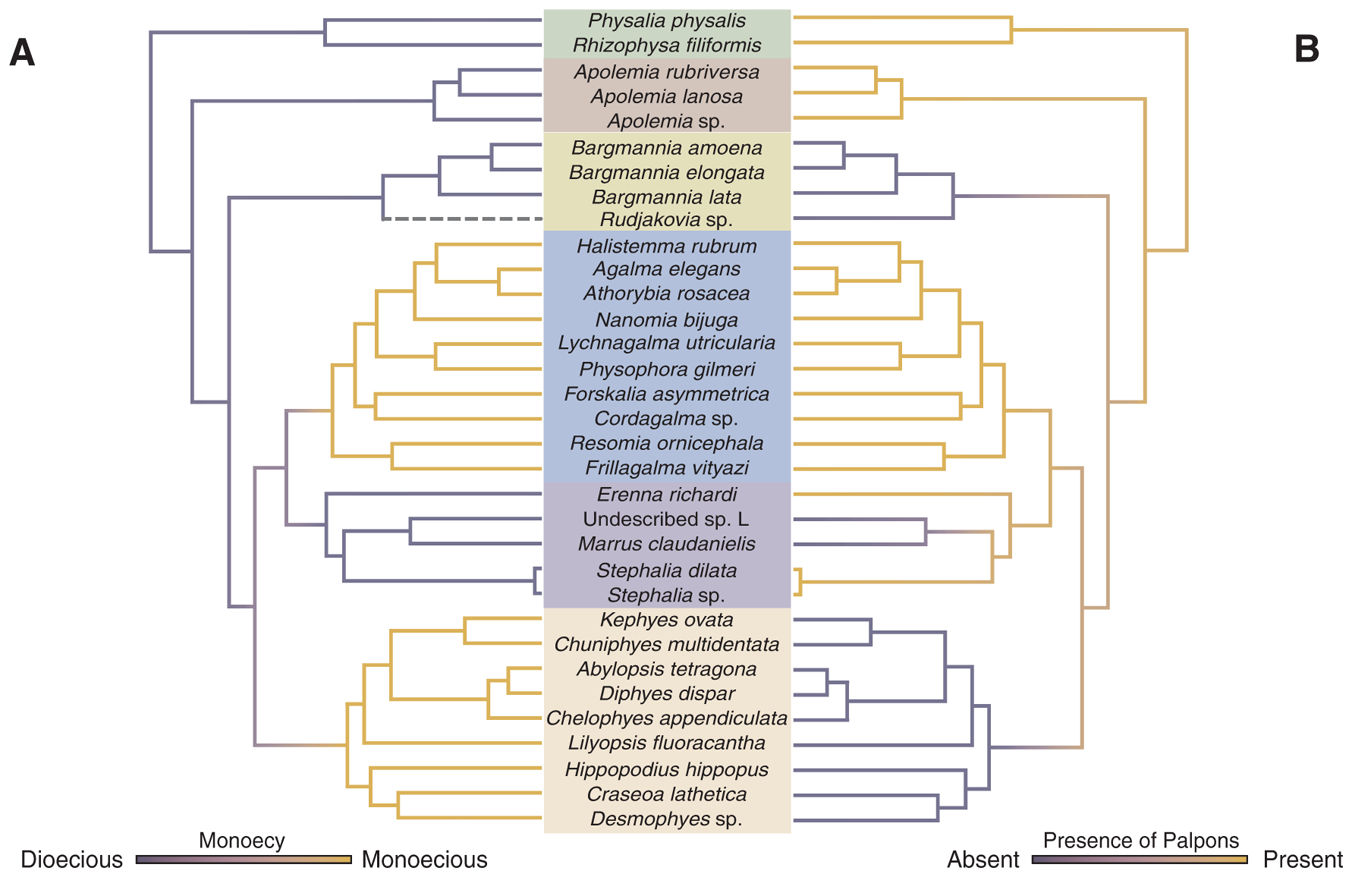
Stochastic mapping reconstruction on the ML tree of the evolutionary history of (A) sexual mode, whether a colony is monoecious or dioecious and (B) presence/absence of palpons (modified reduced gastrozooids). The color gradients show the reconstructed probability estimate of the discrete character states along the branches. Intermediate values reflect uncertainty. The grey dashed branch leading to *Rudjakovia* sp. indicates that the sexual mode of this species is unknown. (For interpretation of the references to colour in this figure legend, the reader is referred to the web version of this article.)

Ancestral character state reconstructions favor the hypothesis that monoecy arose twice (Figs. 5A and S13), once in Calycophorae and once in Clade A. This is consistent with differences in the arrangements of male and female gonophores in the two clades. In Clade A, male and female zooids are found within the same cormidium (a single reiterated sequence of zooids along the stem, see Fig. 2). In these species, the male and female zooids are placed at different but well defined locations within the cormidium. Meanwhile in calycophorans, each cormidium bears either male or female gonophores. In this form of monoecy, the male and female cormidia can either occur in an alternating pattern, or there can be several male or female cormidia in a row. In either case, male and female zooids are found at the same corresponding locations within the cormidia. One known exception to this can be found in abylid calycophorans, where both male or female gonophores may be found within the same eudoxid (Carré, 1967). In sum, homoplasy in sexual system evolution along with variation in the spatial arrangement of gonophores within a colony suggest that siphonophores have evolved different ways to be monoecious. The sexual system and cormidial arrangement of *Rudjakovia* is undescribed, although preliminary observations suggest that this species may be monoecious and that monoecy arose a third time in the Pyrostephidae. A detailed re-description of *Rudjakovia* would help clarify this.

Both Calycophorae and Clade A have a large proportion of shallow water species (see Section 3.6), suggesting that there may be an association between habitat depth and sexual mode. Similar independent transitions from gonochorism (separate sex) to hermaphroditism (both sexes in the same individual) have been identified in shallow-water scleractinian corals (Anthozoa, Cnidaria) (Kerr et al., 2011). To test this hypothesis, a more extensive taxon sampling of the Calycophorae is needed.

Within Calycophorae there are additional variations of the sexual mode: in *Sulculeolaria* (not included in this phylogeny) colonies appear to present a single sex at a time. However they are monoecious and protandrous, with female gonophores developing after the release of male gonophores (Carré, 1979). Environmental influences may also play a role in determining the expressed sex. Colonies of the calycophoran *Chelophyes appendiculata* collected in the field always bear both male and female gonophores, whereas when kept in culture only gonophores of one sex are maintained (Carré and Carré, 2000). This suggests a high plasticity of the sexual state in some calycophoran taxa and underlines the need for caution when evaluating the state of this character in rarely collected species.

### 3.3. The evolution of zooid types

One of the most striking aspects of siphonophore biology is their diversity of unique zooid types (Beklemishev, 1969; Cartwright and Nawrocki, 2010). For example, *Forskalia* and other physonects have at least 5 basic zooid types (nectophore, gastrozooid, palpon, bract, and gonophore), and in some species, there can be nine zooid subtypes (4 types of bract, male & female gonophores) (Pugh, 2003). Here we reconstruct the evolutionary origins of several zooid types on the present transcriptome-based tree (Fig. 4).

Nectophores (Fig. 2) are non-reproductive propulsive medusae. In Codonophora, the nectophores are localized to a region known as the nectosome (Fig. 2B), which has its own growth zone, and they are used for coordinated colony-level swimming. Planktonic cystonects like *Bathyphysa sibogae* and *Rhizophysa filiformis* (Fig. 1A) instead move through the water column using repeated contraction and relaxation of the stem, and in the case of *B. sibogae*, use modified flattened gastro-zooids with wings (called ptera) to increase surface area and prevent colony sinking (Biggs and Harbison, 1976). Nectophores are also present within the gonodendra (reproductive structures) of cystonects, and are thought to propel the gonodendra when they detach from the colony (Totton, 1960, 1965). It is not clear whether the nectophores found within the siphosome of the cystonects are homologous to the nectophores borne on the nectosome of codonophorans. Similarly, the homology of the special nectophore associated with gonophores of the calycophoran *Stephanophyes superba* is also unclear (Chun, 1891). In this study, we only consider the evolution of the nectosome, and not the presence/absence of nectophores. The present analyses, as well as the analyses of Dunn et al. (2005), are consistent with a single origin of the nectosome (Figs. S5 and S17).

Within the nectosome, the nectophores can be attached along the dorsal or ventral side of the stem, following the orientation framework of Haddock et al. (2005). The apparent placement of the nectophores on opposite sides of the nectosome occurs through twisting of the stem during development. Our ancestral reconstructions for this character (Figs. S7 and S19) suggest that ventral attachment of nectophores was the ancestral state in Codonophora, and that dorsal attachment has independently evolved twice – once along the stem of Agalmatidae and once along the stem of Pyrostephidae. The functional implication of dorsal vs. ventral attachment is not clear.

Bracts are greatly reduced zooids unique to siphonophores, where they are only present in Codonophora (Fig. 4). Bracts are functional for protection of the delicate zooids and to help maintain neutral buoyancy (Jacobs, 1937). Some calycophorans are able to actively exclude sulfate ions in their bracts to adjust their buoyancy along the colony (Bidigare and Biggs, 1980). Bracts were lost in Hippopodiidae, some clausophyids, *Physophora hydrostatica* (Fig. 1K), and in *Gymnopraia lapislazula*. These patterns of loss are not captured in this study, as most of these species are not included in the present phylogeny. In species without bracts, other zooids appear to fulfill the roles of buoyancy control and protection. In *P. hydrostatica*, enlarged palpons surround all other si-phosomal zooids and move in a coordinated manner to inflict a powerful sting (Totton, 1965). While in *Hippopodius hippopus* the nectophores play a role in maintaining neutral buoyancy and possibly also in defense, by bioluminescing and blanching in response to stimuli (Fig. 1C shows the blanching of nectophores) (Bassot et al., 1978).

Palpons are typically defined as modified reduced gastrozooids (Mackie et al., 1987). In many species palpons are thought to play a role in digestion and circulation of the gastrovascular fluid, while other species may use them for defense (e.g *Physophora*) or sensory functions (Totton, 1965). Palpons are subcategorised based on their location -palpons that are associated with gonodendra are termed gonopalpons (typically with a reduced tentacle, called a palpacle); palpons found along the stem of the siphosome are termed palpons (typically having a palpacle); and palpons found along the stem of the nectosome are termed nectosomal palpons (as in *Apolemia*) (Siebert et al., 2013; Totton, 1965). It is not clear how structure and function differs among different palpon subtypes, and a detailed histological investigation of palpons found at different locations within species is needed. For this reason, here we only assess the presence or absence of palpons as a category, without assessing subtypes of palpons. This presumes that palpons located at different regions in the colony are derived from other palpons rather than each arising *de novo* by independent modification of gastrozooids, a hypothesis that itself could be challenged upon closer histological examination of palpon diversity.

We reconstruct palpons as present in the common ancestor of siphonophores (Figs. 5B and S14), retained in most species, but lost three times independently in the branches leading to *Bargmannia* and *Rudjakovia* sp., in calycophorans, and in *Marrus claudanielis* and Undescribed sp. L. It remains to be clarified if small buds associated with nectophores within the nectosome of *Bargmannia* species (Dunn, 2005) actually correspond to reduced palpons. The pyrostephid *Pyrostephos vanhoeffeni* (not sampled) has modified tentacle-less palpons (termed oleocysts), but the relationship between this species and *Rudjakovia* sp. is not yet known, so the exact patterns of loss within *Pyrostephidae* (here provisionally including *Rudjakovia* sp.) remain unclear. Within the calycophorans, one species *Stephanophyes superba* (not included in this phylogeny) has polyp-like zooids that have been described as palpons (Totton, 1965), but the exact identity of this zooid is not clear and needs further morphological examination.

### 3.4. The gain and loss of the pneumatophore

The pneumatophore (Fig. 2A) is a gas-filled float located at the anterior end of the colony, which helps the colony to maintain its orientation in the water column, and plays a role in flotation in the case of the cystonects (Church et al., 2015b; Mackie, 1974; Totton, 1965). It is not a zooid, as it is not formed by budding but by invagination at the aboral end of the planula during early development (Carré, 1969; Garstang, 1946; Leloup, 1935). Recent descriptions of the neural arrangement in the pneumatophore of *Nanomia bijuga* suggest it could also gather information on relative pressure changes (and thus depth changes), helping regulate geotaxis (Church et al., 2015b). The ancestral siphonophore had a pneumatophore (Fig. 2B), since both cystonects and all “physonects” possess one (Fig. 4). The pneumatophore was lost in Calycophorae and never regained in that clade. Calycophorans rely on the ionic balance of their gelatinous nectophores and bracts to retain posture and neutral buoyancy (Mackie, 1974).

### 3.5. The gain and loss of tentilla

Gastrozooids (specialized feeding polyps) have a single tentacle attached to the base of the zooid that is used for prey capture (with the exception of *Physalia physalis*, which has separate zooids for feeding and prey capture, and rhodalids, where some tentacles are used to anchor to the substrate and do not participate in feeding). As in other cnidarians, stinging capsules, arranged in dense batteries of nematocysts, play a critical role in prey capture. In many siphonophore species these batteries are found in side branches of the tentacle, termed tentilla (Fig. 2A). Outside of Siphonophora, most hydrozoans bear simple tentacles without side branches. It is still an open question whether the common ancestor of Siphonophora had tentilla. The only siphonophore species regarded as lacking tentilla are *P. physalis*, *Apolemia* spp. (Fig. 1H), and *Bathyphysa conifera* (Fig. 1B). Since *B. conifera* is the only member of the Rhizophysidae (and of the *Bathyphysa* genus) lacking tentilla, we assume this is a case of secondary loss. When we reconstruct the evolution of this character on the current phylogeny, 70% of simulations support a common ancestor bearing tentilla, with two independent losses leading to *Physalia* and *Apolemia* (Figs. S3 and S15). However, this leaves a 30% support for a simple-tentacled common ancestor followed by 2 independent gains of tentilla in the branches leading to Rhizophysidae and non-apolemiid codonophorans.

How we define absence of tentilla, especially for *Physalia physalis*, is also important. The tentacles of this species, when uncoiled, show very prominent, evenly spaced, bulging buttons which contain in the ectoderm functional nematocytes (carrying mature nematocysts) used by the organism for prey capture (Hessinger and Ford, 1988; Totton, 1960). Siphonophore tentilla are complete diverticular branchings of the tentacle ectoderm, mesoglea, and gastrovascular canal (lined by endoderm). *Physalia*’s buttons enclose individual fluid-filled chambers connected by narrow channels to the tentacular canal, lined by endoderm (Bardi and Marques, 2007). This suggests they are not just ectodermal swellings, but probably reduced tentilla. When we define *P. physalis* as tentilla bearing, the results for the character reconstruction lead to a more robust support for a tentilla-bearing common ancestor followed by independent losses of tentilla in the branch leading to Apolemiidae (Figs. S4 and S16), and in *Bathyphysa conifera*. The application of phylogenetic methods to the evolution of tentillum morphology would be a crucial step towards understanding the evolution of these structures, and their relationship with the feeding ecology of siphonophores.

### 3.6. The evolution of vertical habitat use

Siphonophores are abundant predators in the pelagic realm, ranging from the surface (*Physalia physalis*) to bathypelagic depths (Figs. 4, S8, S20) (Mackie et al., 1987; Mapstone, 2014). The depth distribution of siphonophore populations is not always static, as some species are known to be vertical migrators, although this is within a relatively narrow depth range (< 100 m) (Pugh, 1984). Some species such as *Nanomia bijuga* exhibit synchronous diel migration patterns (Barham, 1966). Using the present phylogeny, we reconstructed the median depth changes along the phylogeny under a Brownian Motion model (Figs. S8 and S20), which had the strongest AICc support (compared to non-phylogenetic distributions, and to Ohrnstein-Uhlenbeck). This model indicates a mesopelagic most recent common ancestor, with several independent transition events to epipelagic and bathypelagic waters. There was only a single transition to benthic lifestyle on the branch of Rhodaliidae, and a single transition to a pleustonic lifestyle on the branch of *P. physalis*. There is evidence that habitat depth is conserved within some clades, with the exception of Calycophorae which have diversified across the water column (Figs. S8 and S20). Under the ML topology, depth appears to be phylogenetically conserved in Euphysonectae after the split between Clade A (shallow-living species) and Group B (deep-dwelling species) (Fig. S8), while under the BI-CAT topology, a mesopelagic common ancestor is predicted, with a transition to epipelagic waters in Clade A (Fig. S20); however several shallow-living species that likely belong in Group B were not included in this analysis. The present sampling is also not sufficient to capture significant variation in depth distributions between closely related species. Previous studies have shown that many species that are collected at the same locality are found to occupy discrete, largely non-overlapping depth distributions, including between species that are closely related (Pugh, 1974). This suggests that vertical habitat use is more labile than it appears and may be an important mechanism in siphonophore ecology. The observed variation in depth distribution could be attributed to any of the correlated environmental variables (i.e. temperature, chlorophyll, oxygen). Temperature has been hypothesized to impose physiological limits to the dispersal of some clausophyid siphonophores (Grossmann et al., 2015). Since most of our specimens were sampled only in the Monterey Bay region, our analyses of the local oceanographic and depth distribution data cannot disentangle the effects of these different variables on the vertical distributions.

This reconstruction (Figs. S8 and S20) only included depths recorded using an ROV, thus it excludes many other independent colonizations of the epipelagic habitat. The ROV observations are reliable below 200 m, and no quantitative measurements were made on SCUBA dives. Species such as *Nanomia bijuga*, *Hippopodius hippopus*, *Athorybia rosacea*, *Diphyes dispar*, and *Chelophyes appendiculata* are often encountered blue water diving less than 20 m from the surface (Fig. 4). We also reconstructed the median depth changes along the phylogeny using median depths of 20 m for all species collected by SCUBA diving or via a shallow trawl (Figs. S9 and S21), and still find support for a mesopelagic ancestor. It should be noted, however, that *H. hippopus* and *C. appendiculata* were both collected in the bay of Villefrance-sur-mer, France, where an upwelling is known to bring deeper species closer to the surface (Nival et al., 1976). Additionally, while we are confident about many of the species IDs in the VARS dataset, it is difficult to distinguish *Kephyes ovata* and *K. hiulcus* from images alone and the distribution likely includes data points from both species. *Halistemma rubrum* distributions were obtained from cruises in the Gulf of California, where the only *Halistemma* species collected by ROV is *H. rubrum*. Where we could not be certain of species identifications in the VARS dataset, we only included a few data points from specimens that were collected and identified.

## 4. Conclusions

Using phylogenomic tools we were able to resolve deep relationships within Siphonophora with strong support. We identify the clade Euphysonectae as the sister group to Calycophorae. Our results suggest that monoecy arose at least twice, based both on phylogenetic reconstruction and differences in the way monoecy is realized in different clades. We are unable to fully capture some of the complex patterns of zooid gain and loss within Codonophora, which will require greater taxon sampling and improved morphological understanding of many poorly known species. The improved resolution presented in this study suggests that an important next step in understanding siphonophore evolution will be targeting molecular sampling within Euphysonectae (where we sampled 13 of 62 valid described species that likely belong to the group) and Calycophorae (where we sampled 9 species in a clade of 109 valid described species) to further resolve the internal relationships within these clades.

## Supplementary Material

1

2

3

## Figures and Tables

**Table 1:** A complete list of specimens used in this work, information from already published datasets added where available. New data indicated by Y, blank fields indicate that data were already published. For the species not on SRA, a link to the data is included in supplementary data 1.

New data	Species	Depth (m)	Lat Lon	SRA Number
Y&N	*Nanomia bijuga*	414/387	36.60 N 122.15 W	SRR1548376;SRR1548377;SRR871527
Y	*Bargmannia elongata*	412/805/636/818	36.12 N 122.67 W	SRR1548343–47
Y	*Frillagalma vityazi*	407	36.69 N 122.05 W	SRR1548362;SRR1548363;SRR1548364
Y	*Apolemia rubriversa*	767	36.70 N 122.05 W	SRR1548342
Y	*Chelophyes appendiculata*	3–20	43.696 N, 7.308 E	SRR1548354
Y	*Chuniphyes multidentata*	327	36.79 N 122.00 W	SRR1548355
Y	*Cordagalma* sp	252	36.70 N 122.06 W	SRR1548356
Y	*Erenna richardi*	1044	36.61 N 122.38 W	SRR1548360
Y	*Forskalia asymmetrica*	253	36.80 N 122.00 W	SRR1548361
Y	*Hippopodius hippopus*	3–20	43.69 N 7.315 E	SRR1548371
Y	*Kephyes ovata*	452	36.36 N 122.81 W	SRR1548372
Y	*Lilyopsis fluoracantha*	320	36.69 N 122.04 W	SRR1548373
Y	*Lychnagalma utricularia*	431	36.69 N 122.04 W	SRR1548374
Y	*Marrus claudanielis*	1427	36.07 N 122.29 W	SRR1548375
Y	Undescribed sp. L	1463	36.70 N 122.57 W	SRR1548381
Y	*Desmophyes* sp.	1363	35.48 N 123.64 W	SRR1548358
Y	*Resomia ornicephala*	322	35.48 N 123.86 W	SRR1548382
Y	*Rhizophysa filiformis*	10	27.23 N 110.46 W	SRR1548383
Y	*Stephalia dilata*	3074	35.62 N 122.67 W	SRR1548384
Y	*Apolemia lanosa*	1073	36.70 N 122.08 W	SRR6512857
Y	*Apolemia* sp	461	36.60 N 122.15 W	SRR6512854
Y	*Bargmannia amoena*	1251	36.70 N 122.08 W	SRR6512862
Y	*Bargmannia lata*	1158	36.067 N 122.30 W	SRR6512863
Y	*Rudjakovia* sp	334	36.00 N 122.42 W	SRR6512851
Y	*Stephalia* sp	3255	36.39 N 122.67 W	SRR6512855
Y	*Physophora gilmeri*	242	36.36 N 122.40 W	SRR6512853
Y	*Halistemma rubrum*	313	24.68 N 109.90W	SRR6512852
Y	*Athorybia rosacea*	3–20	22.92 N 108.36 W	SRR6512856
Y	*Diphyes dispar*	3–20	35.93 N 122.93 W	SRR6512850;SRR6512858–61;SRR6512864;SRR6512867–68
	*Agalma elegans*	3–20	35.56 N 122.55 W	SRR6512865;SRR6512866
	*Physalia physalis*	0	13.831 N 129.943 W	SRR871528
	*Abylopsis tetragona*	3–20	43.696 N, 7.308 E	SRR871525
	*Aegina citrea*		36.697177 N 122.054095 W	SRS893439
	*Aiptasia pallida*			SRR6967; SRR6967; SRR6967
	*Alatina alata*		12.151891 N 68.278002 W	SRR1952741
	*Atolla vanhoeffeni*		36.707311 N 122.061062 W	SRR1952729
	*Clytia hemisphaerica*		43.696 N, 7.308 E	N/A
	*Ectopleura larynx*			SRR923510
	*Hydra magnipapillata*			N/A
	*Hydractinia symbiolongicarpus*			SRX474878
	*Nematostella vectensis*			N/A
	*Podocoryna carnea*			SRR1266262
	*Craseoa lathetica*	1530		SRR871529

## Appendix A. Supplementary material

Supplementary data associated with this article can be found, in the online version, at https://doi.org/10.1016/j.ympev.2018.06.030.
