# Supplementary material for: Improved phylogenetic resolution within Siphonophora (Cnidaria) with implications for trait evolution
Source: Mol Phylogenet Evol. Author manuscript; Available in PMC 2019 Oct 1. (PMC6064665; doi:10.1016/j.ympev.2018.06.030)
Supplement: 1 [file NIHMS979652-supplement-1.pdf]

Supplementary Information

Agalma analysis

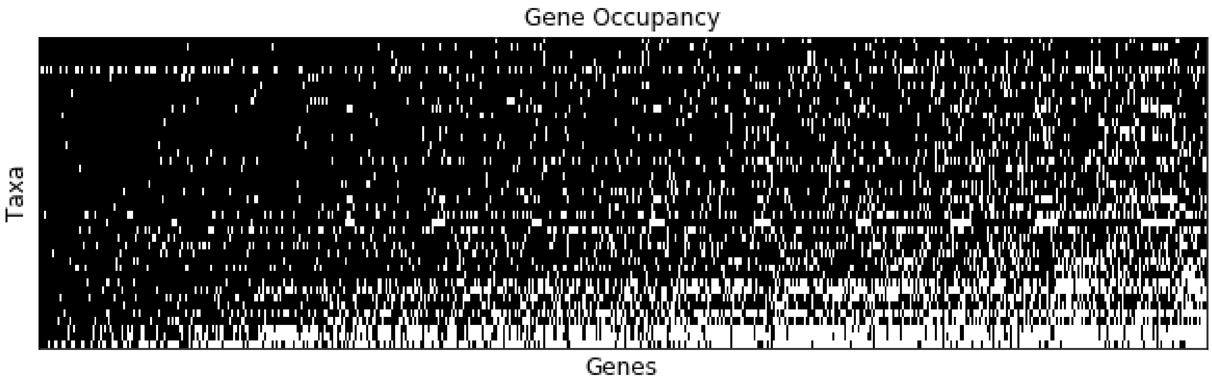

Figure S1: 80% gene occupancy matrix for 41 species across 1,423 genes.

SOWH analysis

	constraint topology	best tree estimated under constraint	test statistic difference in lnL (observed data)	range of null distribution (simulated data)	p-value (confidence interval, n = 100)
monophyletic physonects	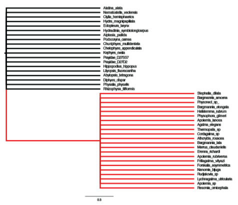	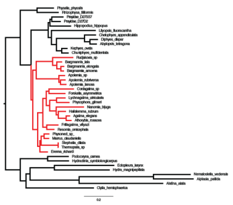	15226.9	$0.10*10^{-3} -0.11*0^{-3}$	<0.01 (0 - 0.03)
monophyletic monoecious	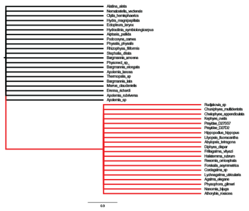	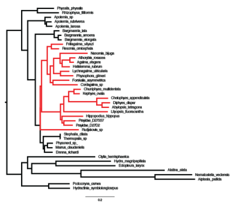	66213	$-6*10^{-5}$ - 0.69	<0.01 (0 - 0.03)

Figure S2: Constrained topologies specified in SOWH testing. Test statistic and p-value for each tree estimated under constraint are given.

Stochastic Character maps

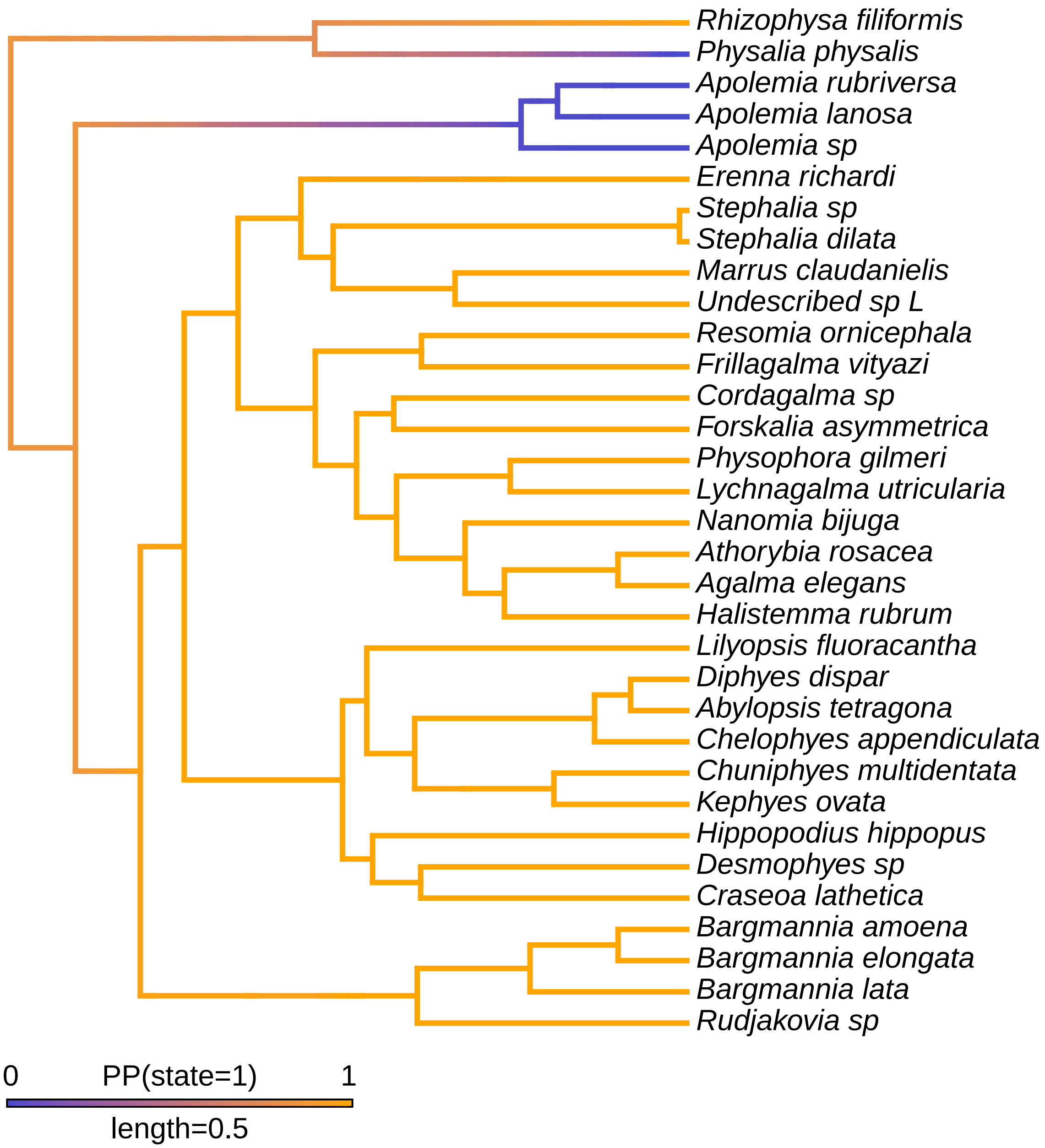

Figure S3: Stochastic character map of presence of tentilla with *Physalia* included as not bearing tentilla.

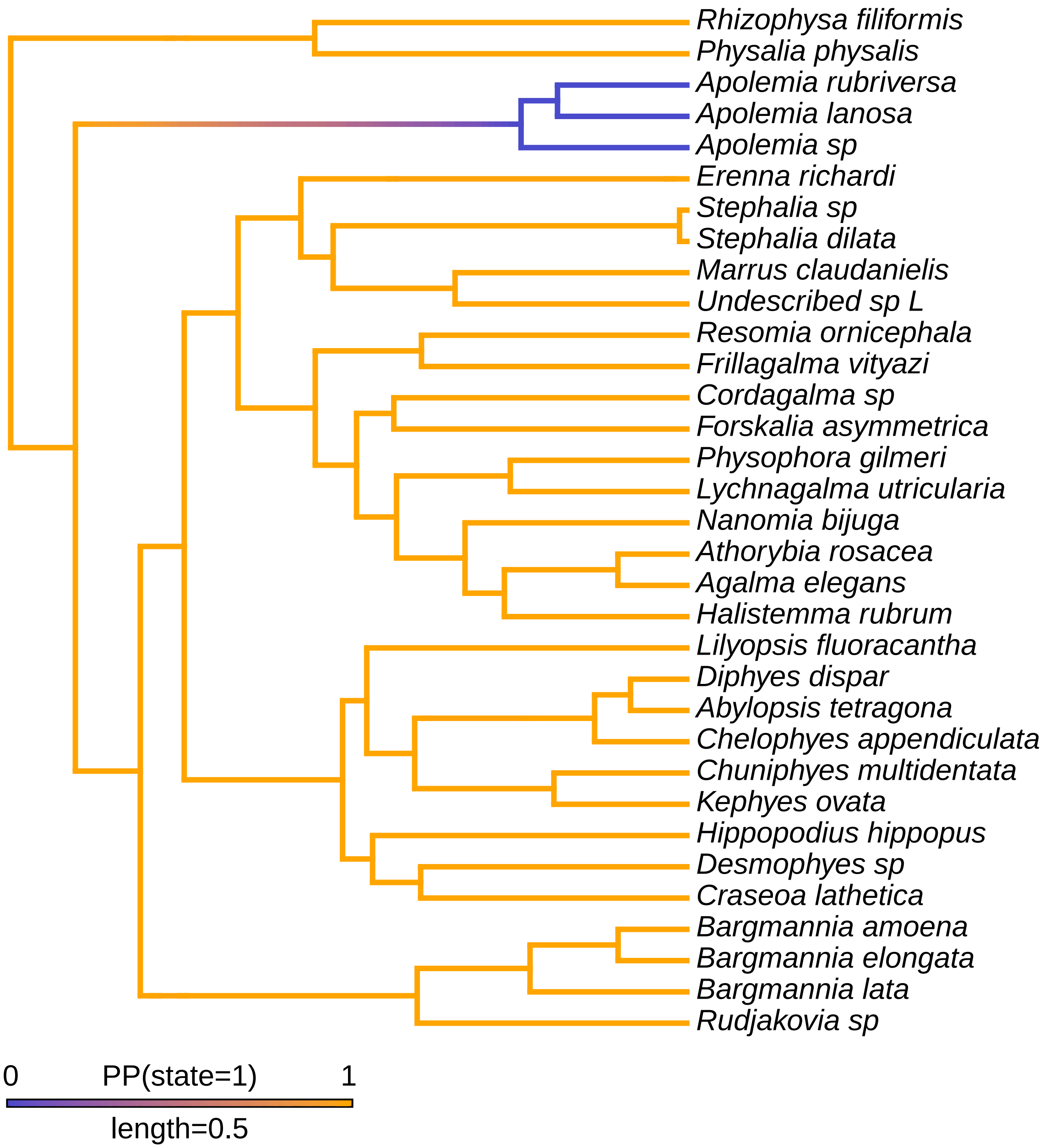

Figure S4: Stochastic character map of presence of tentilla with *Physalia* included as bearing tentilla.

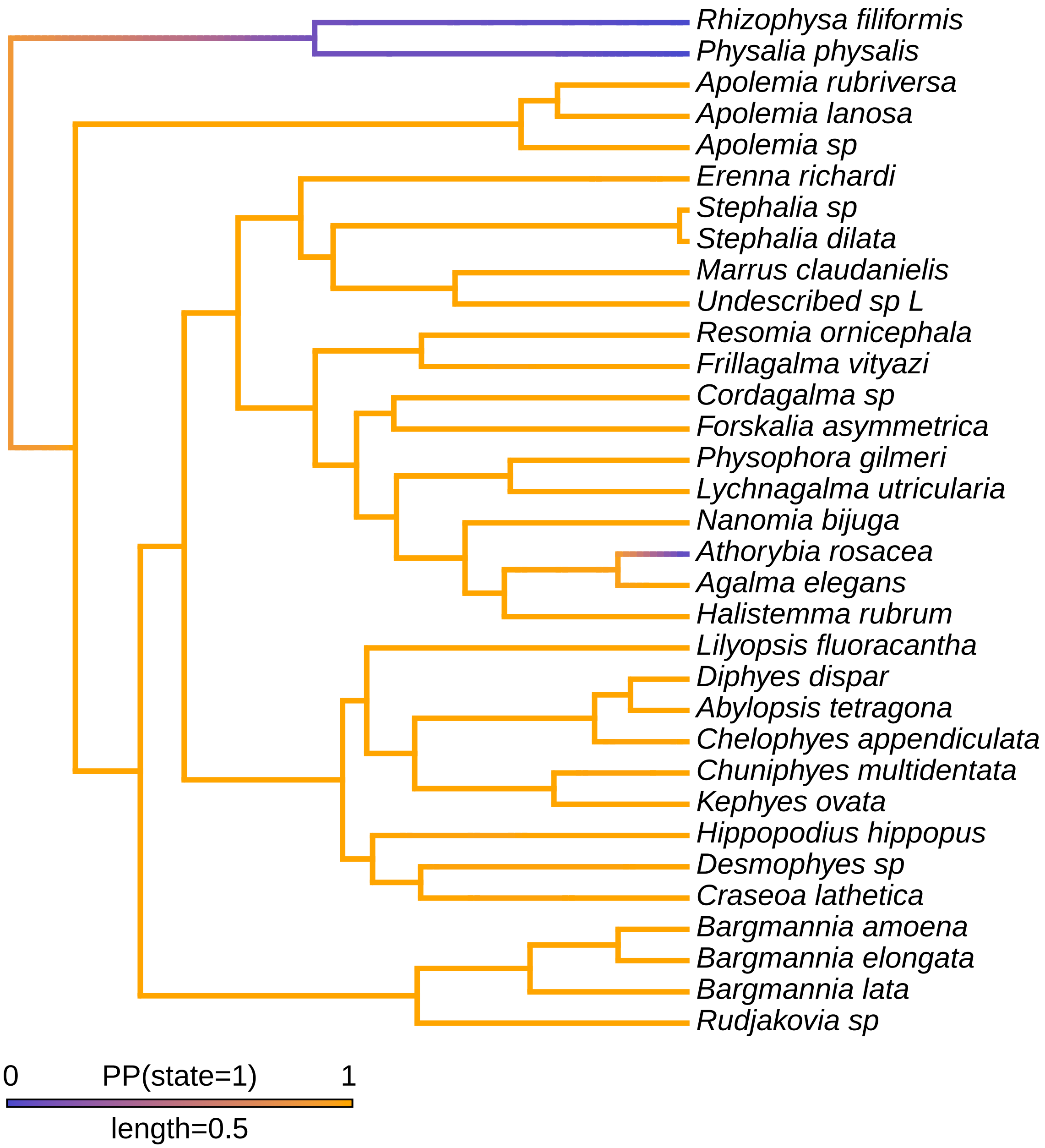

Figure S5: Stochastic character map of presence of nectosome.

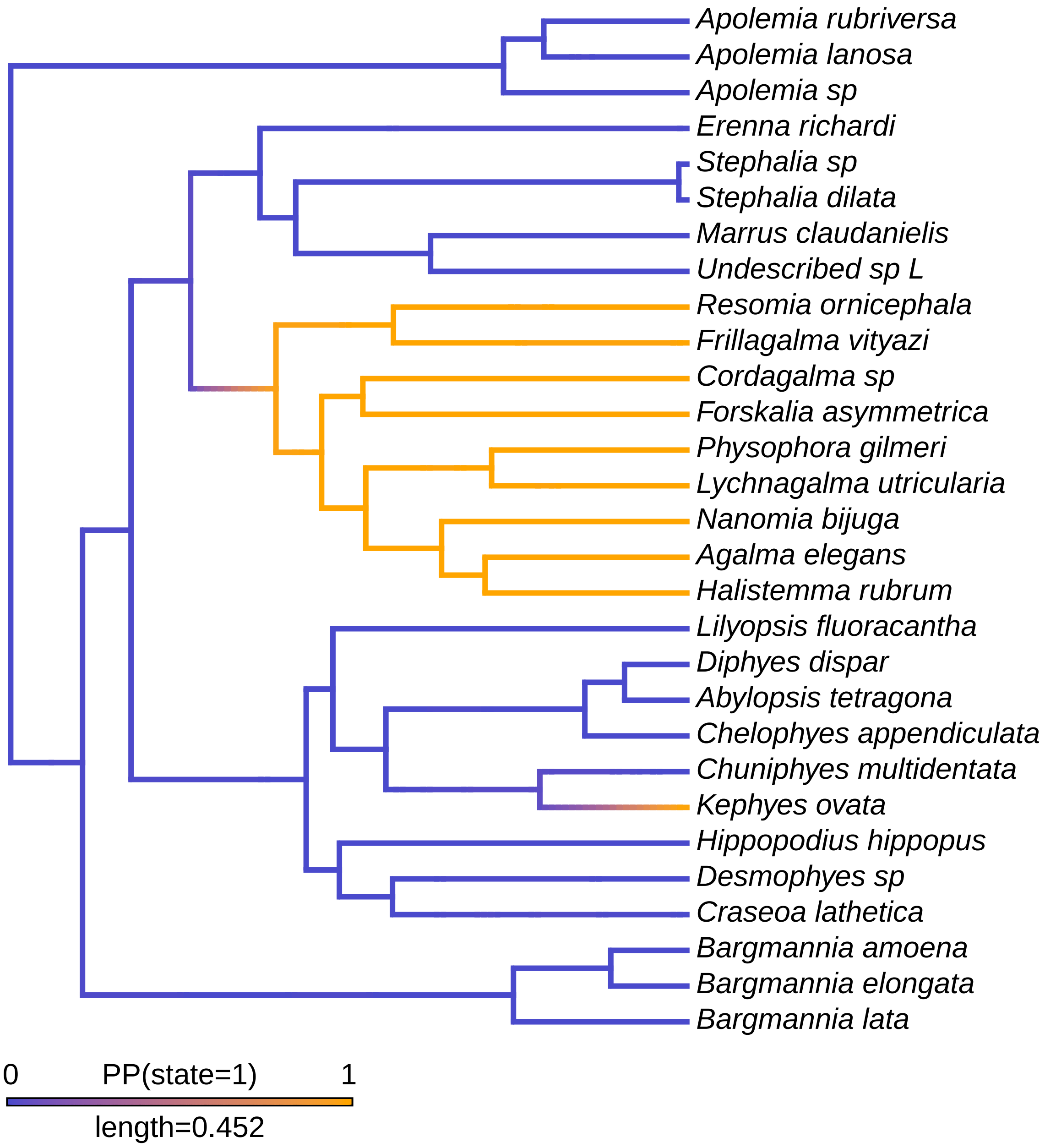

Figure S6: Stochastic character map of presence of a descending mantle canal in the nectophores. Cystonects and Athorybia were excluded as they do not have a nectosome.

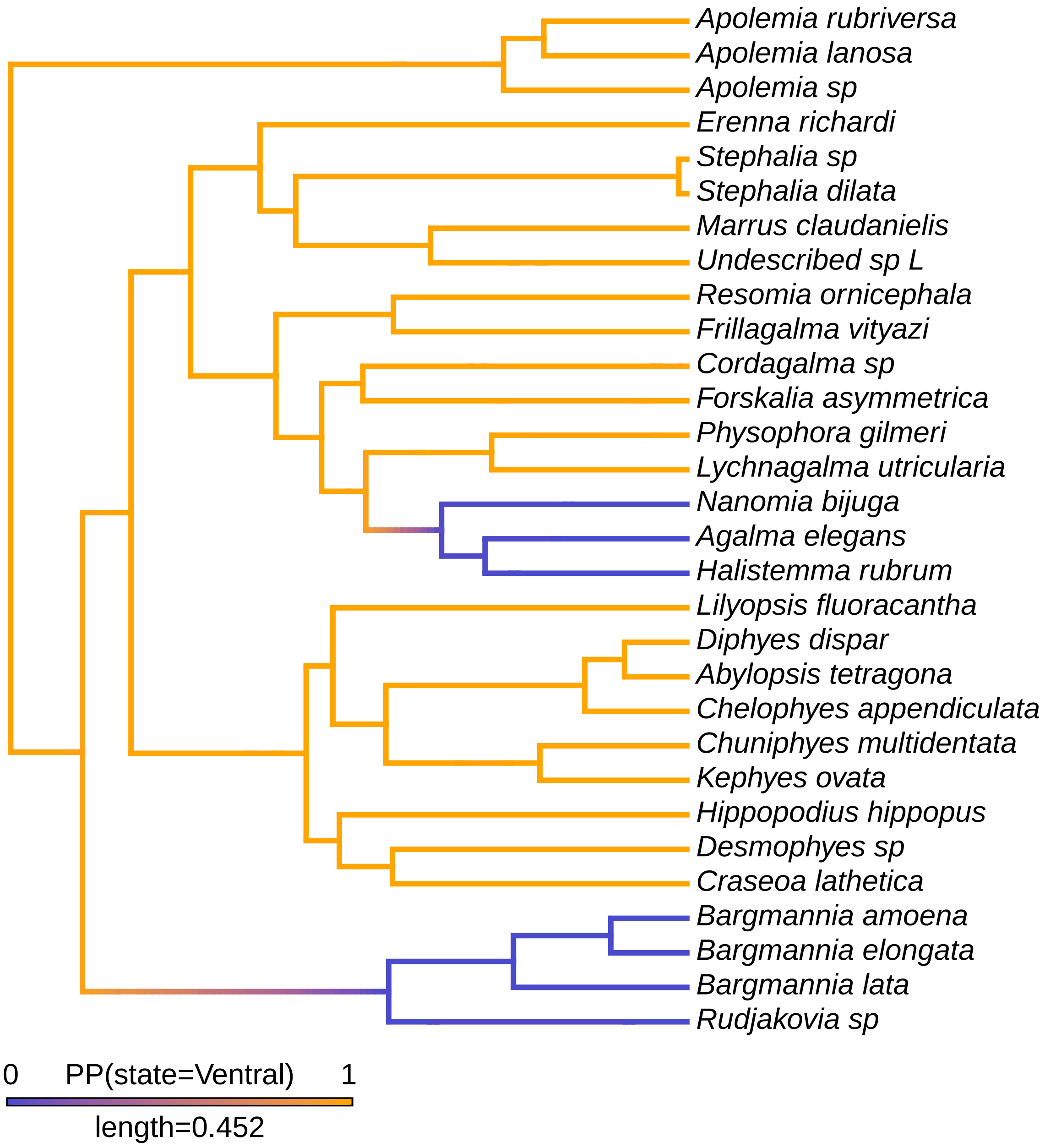

Figure S7: Stochastic character map for the evolution of the position of the nectosome. Cystonects were excluded as they do not have a nectosome.

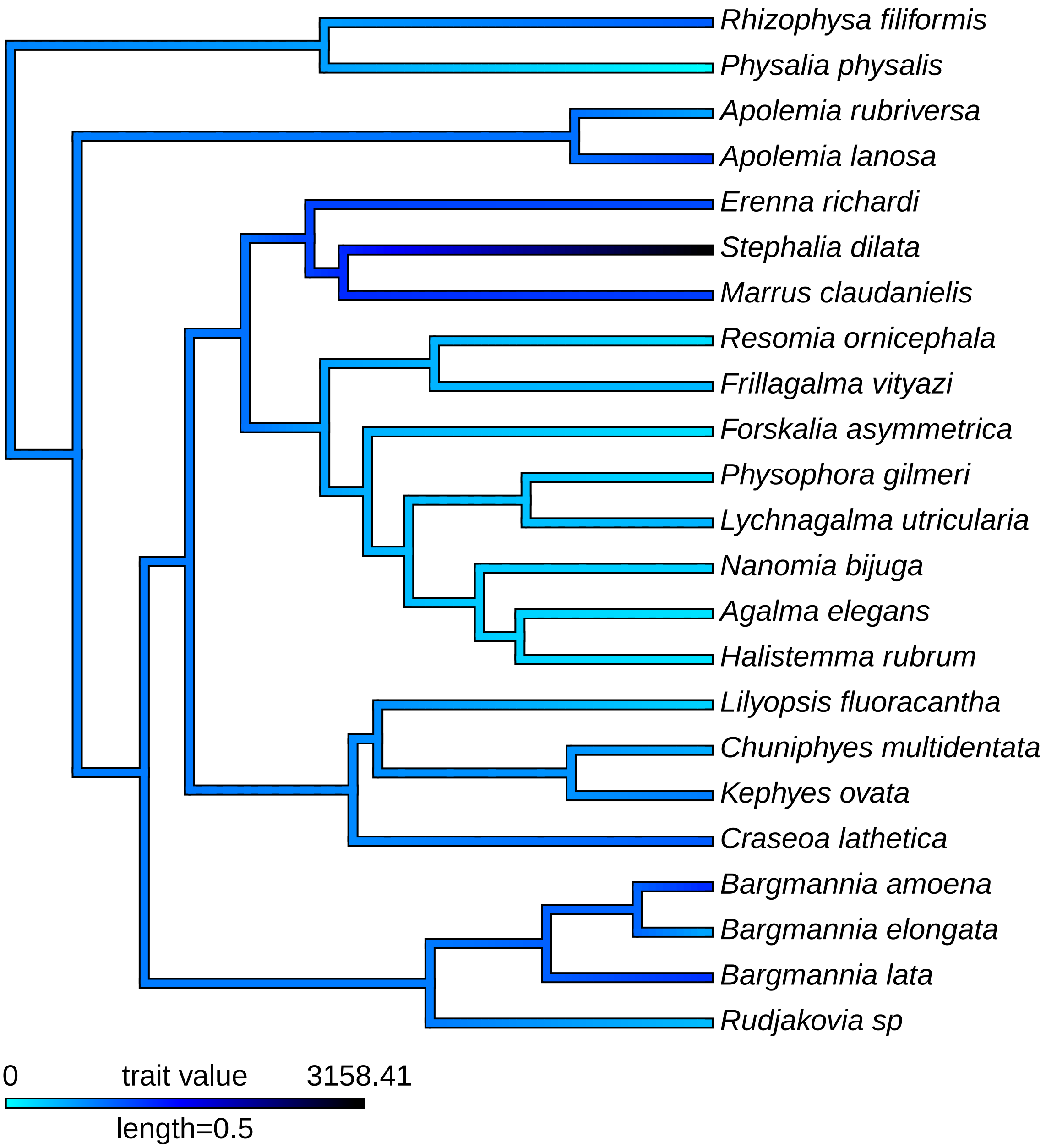

Figure S8: Brownian Motion character map of median depth of species observed with an MBARI ROV.

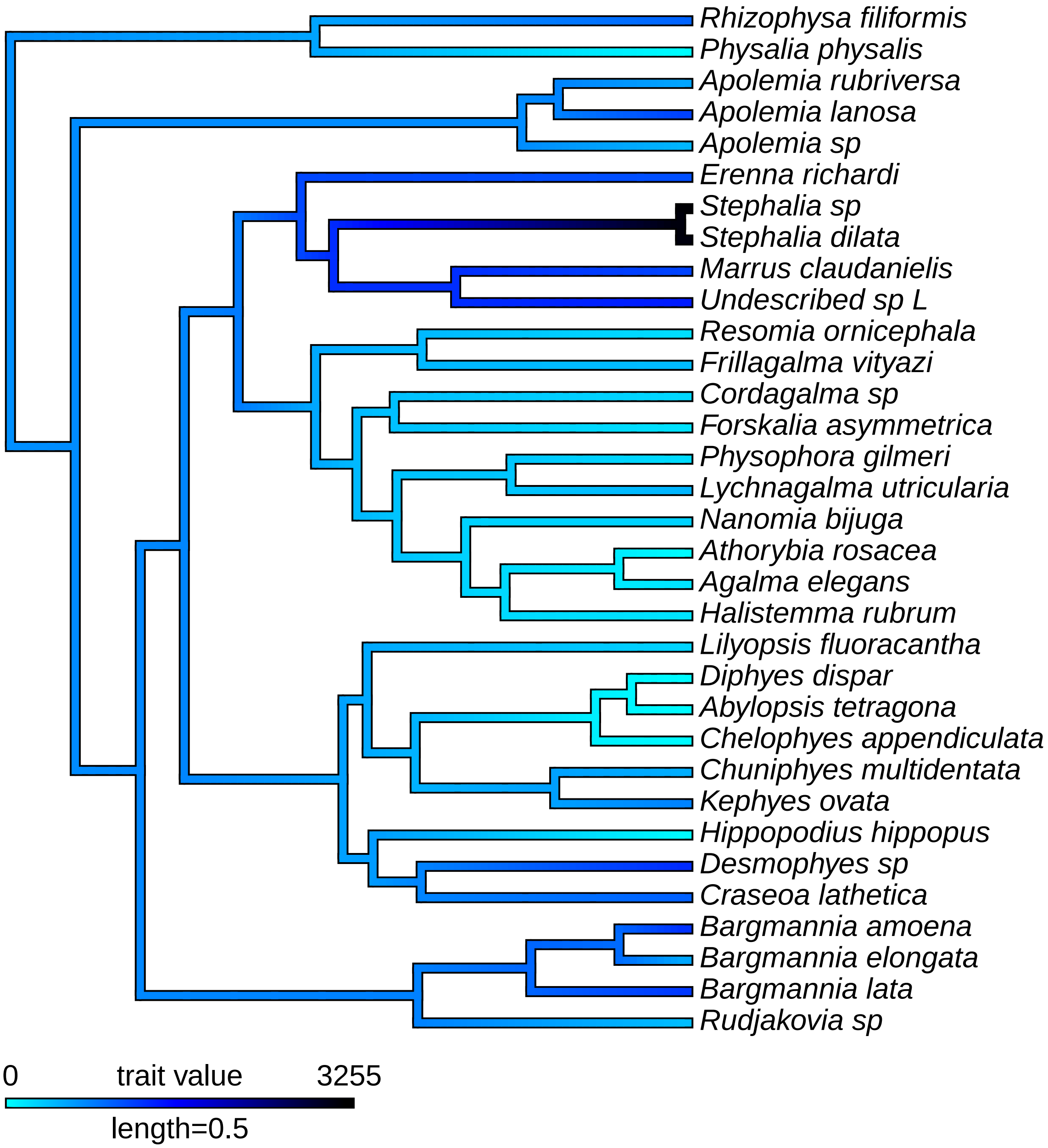

Figure S9: Brownian Motion character map of median depth of species including blue water diving observations.

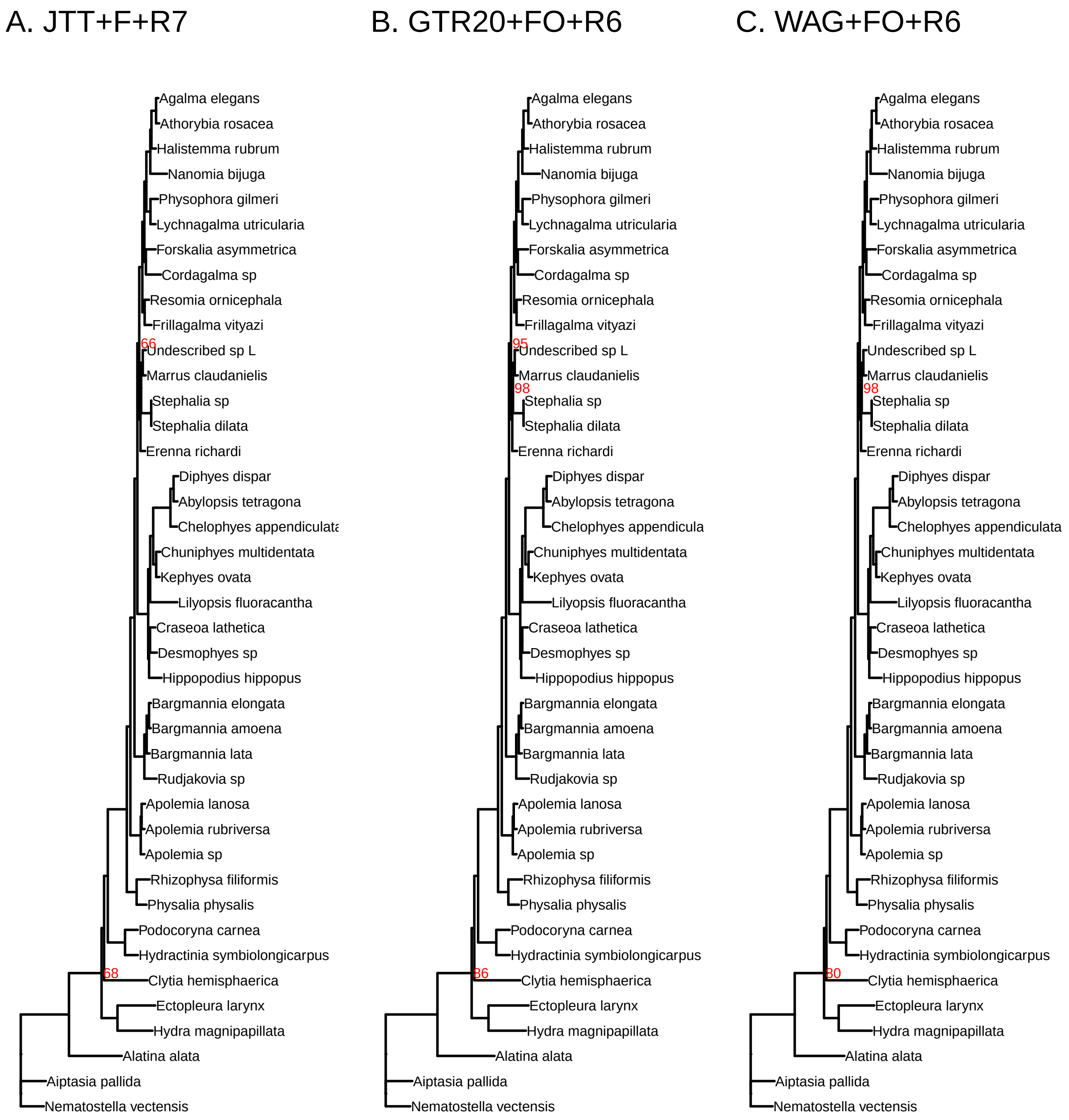

Figure S10: Phylogenies obtained using different models of evolution in IQTree. We used ModelFinder (Kalyaanamoorthy et al., 2017) to identify the model with the best relative model fit. We also present results for the commonly used WAG and GTR models. Unlabeled nodes have support >0.99. A. JTT + Empirically counted frequencies from alignment + FreeRate model with 7 categories (the model identified by ModelFinder as having the best fit). Log likelihood:-8113694.922; AIC:16227609.9565 . B. GTR + Optimized base frequencies by maximum-likelihood + Free rate model with 6 categories. Log likelihood: -8133157.335; AIC:16266530.7277. C. WAG + Optimized base frequencies by maximum-likelihood + Free rate model with 6 categories. Log likelihood: -8156043.772; AIC score: 16312303.6120

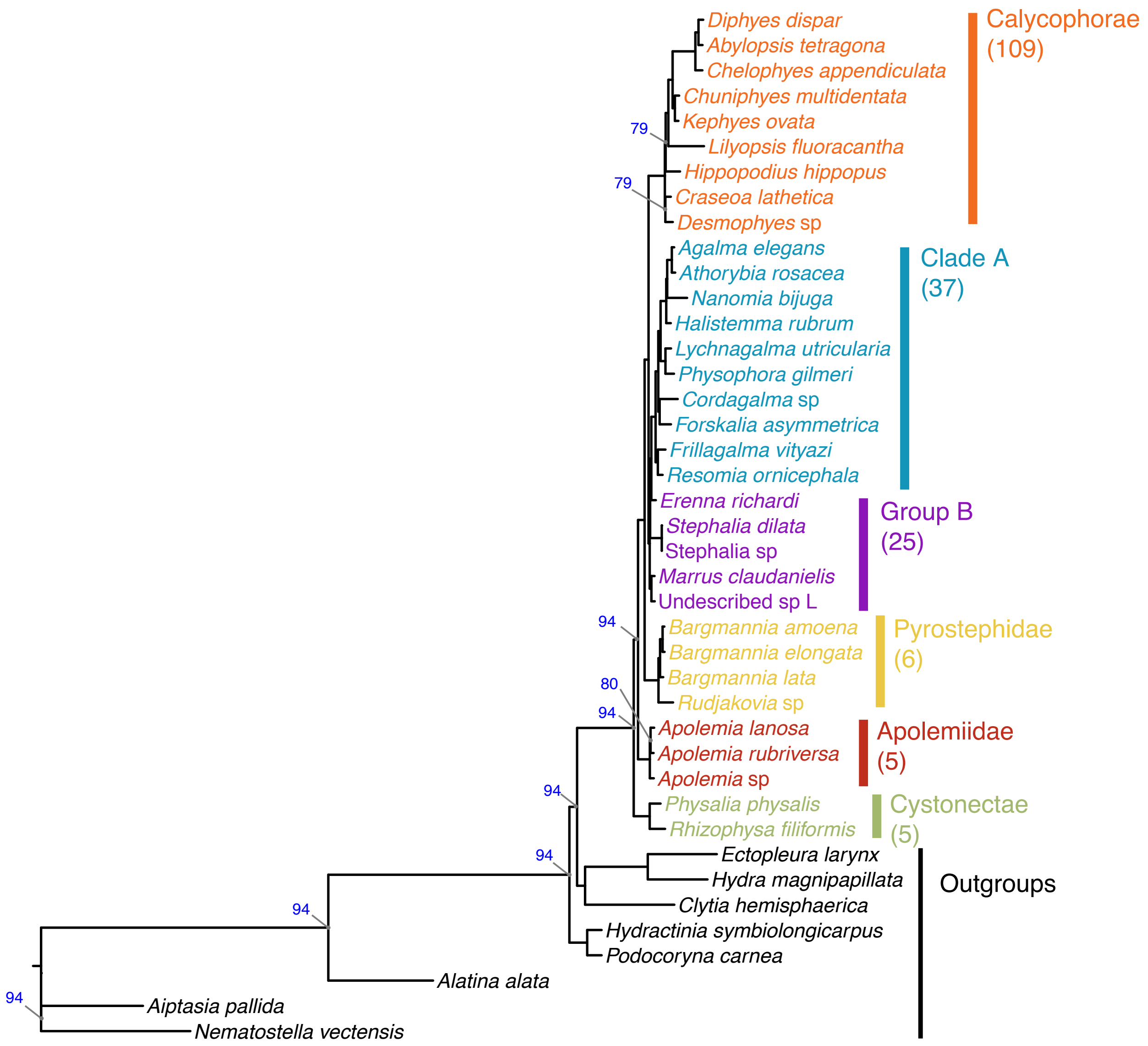

Figure S11: Bayesian (BI) CAT poisson phylogram with bipartition frequencies from the Bayesian posterior distribution of trees. Unlabeled nodes have support >0.99. The numbers of valid described species estimated to be in each clade based on taxonomy are shown below each clade name on the right.

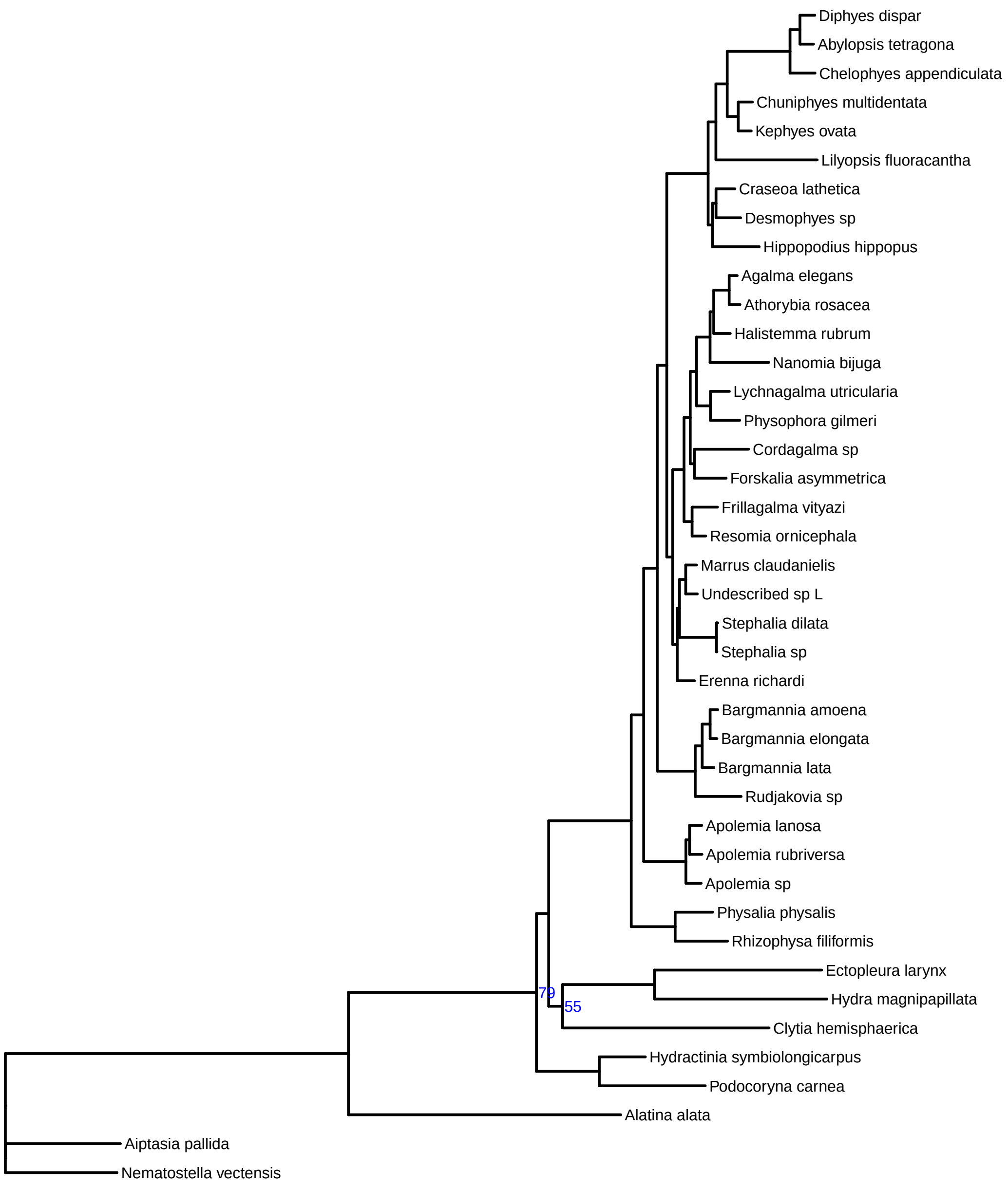

Figure S12: Bayesian (BI) WAG and Gamma phylogram with bipartition frequencies from the Bayesian posterior distribution of trees. Unlabeled nodes have support >0.99.

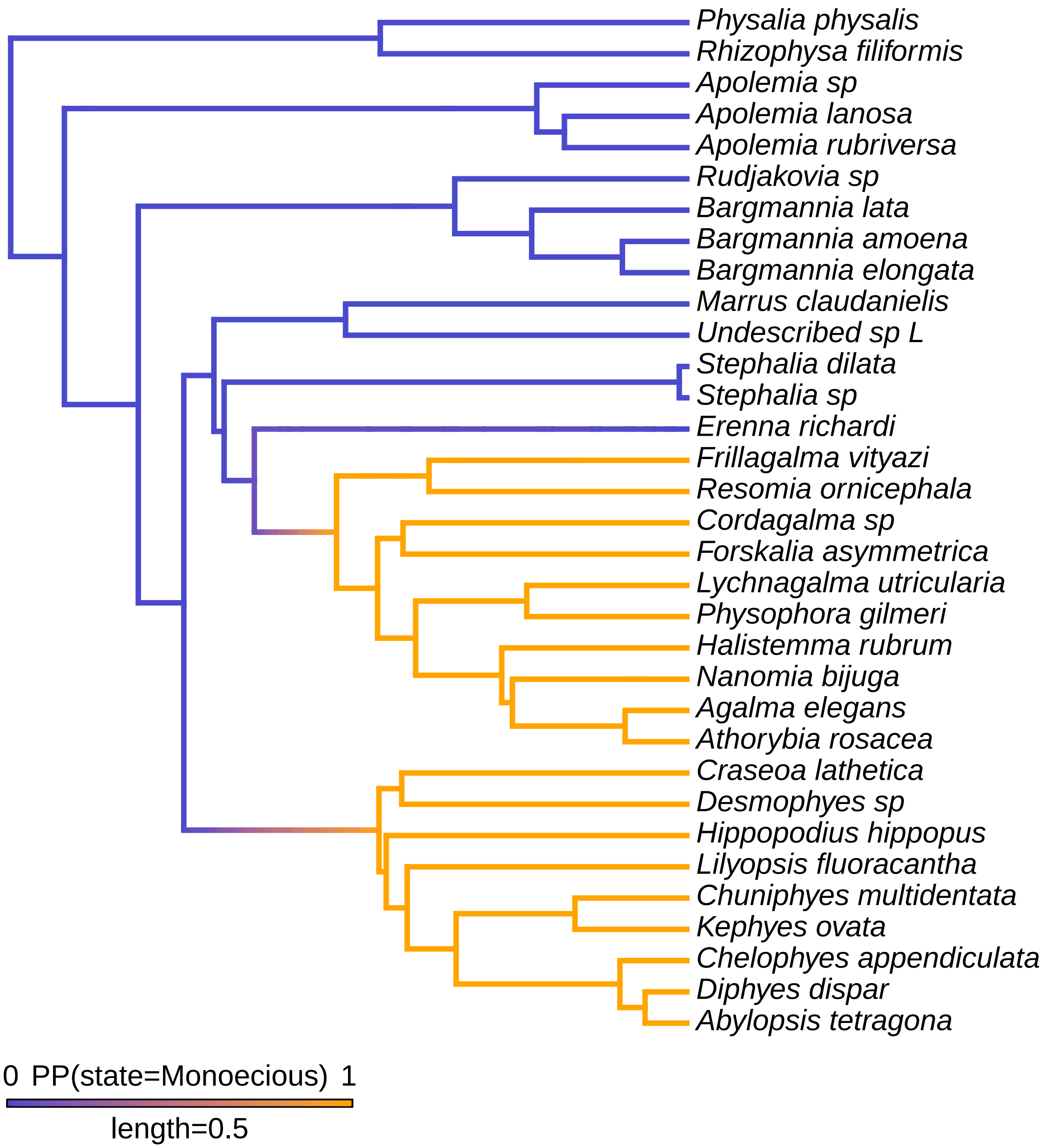

Figure S13: Brownian Motion character map of sexual characters on the consensus Bayesian tree topology.

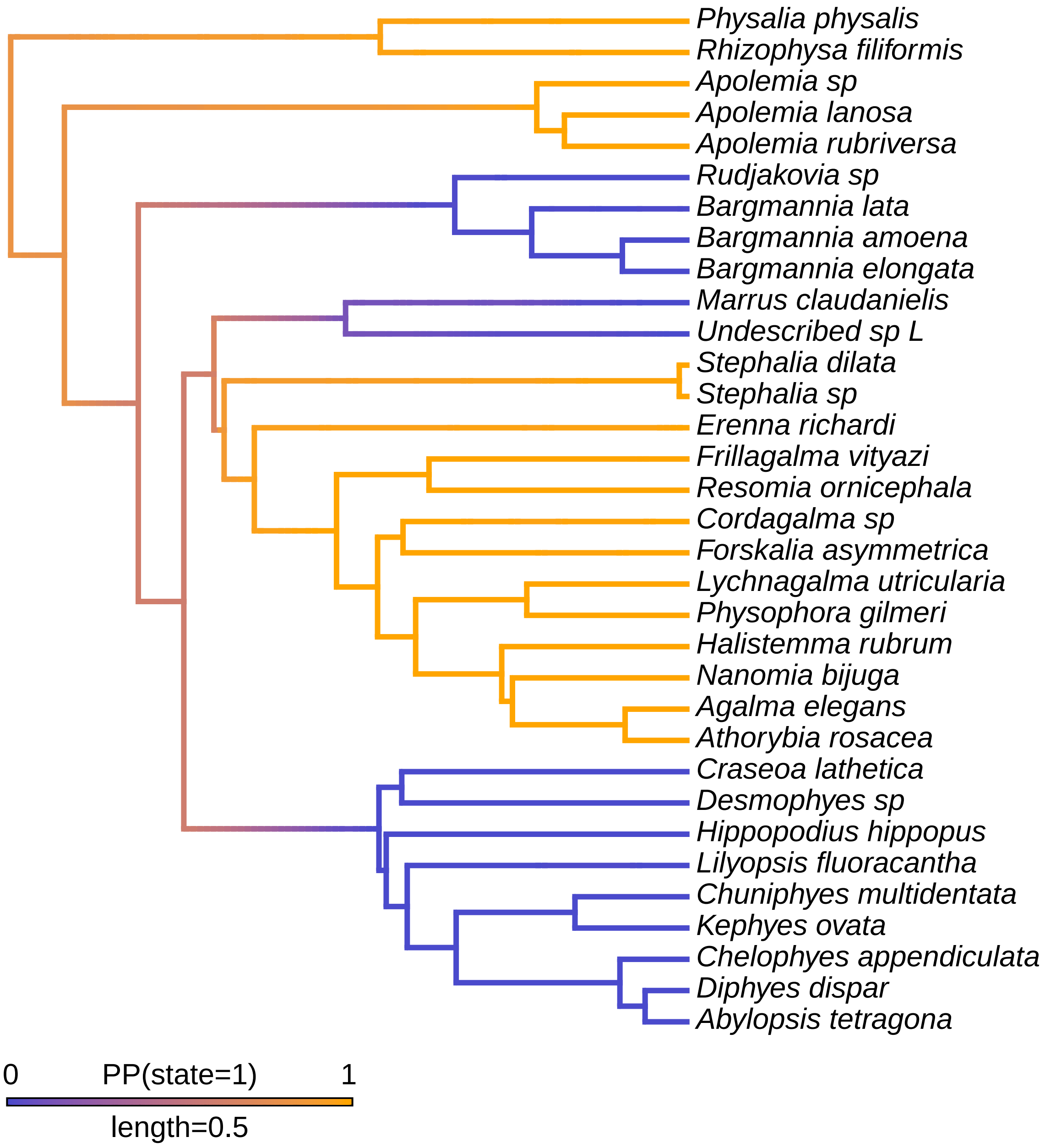

Figure S14: Brownian Motion character map of palpon presence/absence on the consensus Bayesian tree topology.

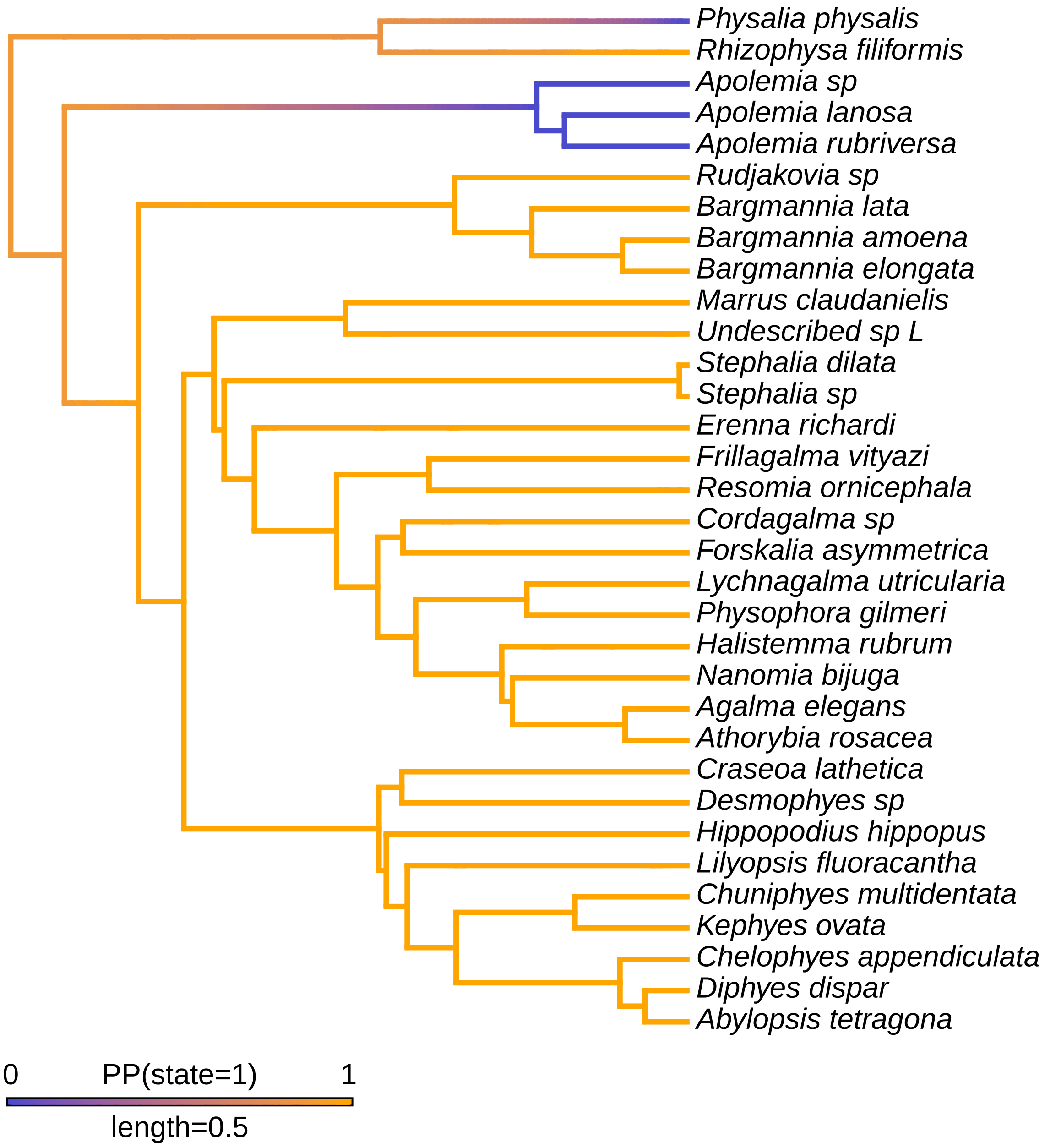

Figure S15: Stochastic character map of presence of tentilla with *Physalia* included as not bearing tentilla, mapped on the consensus Bayesian tree topology.

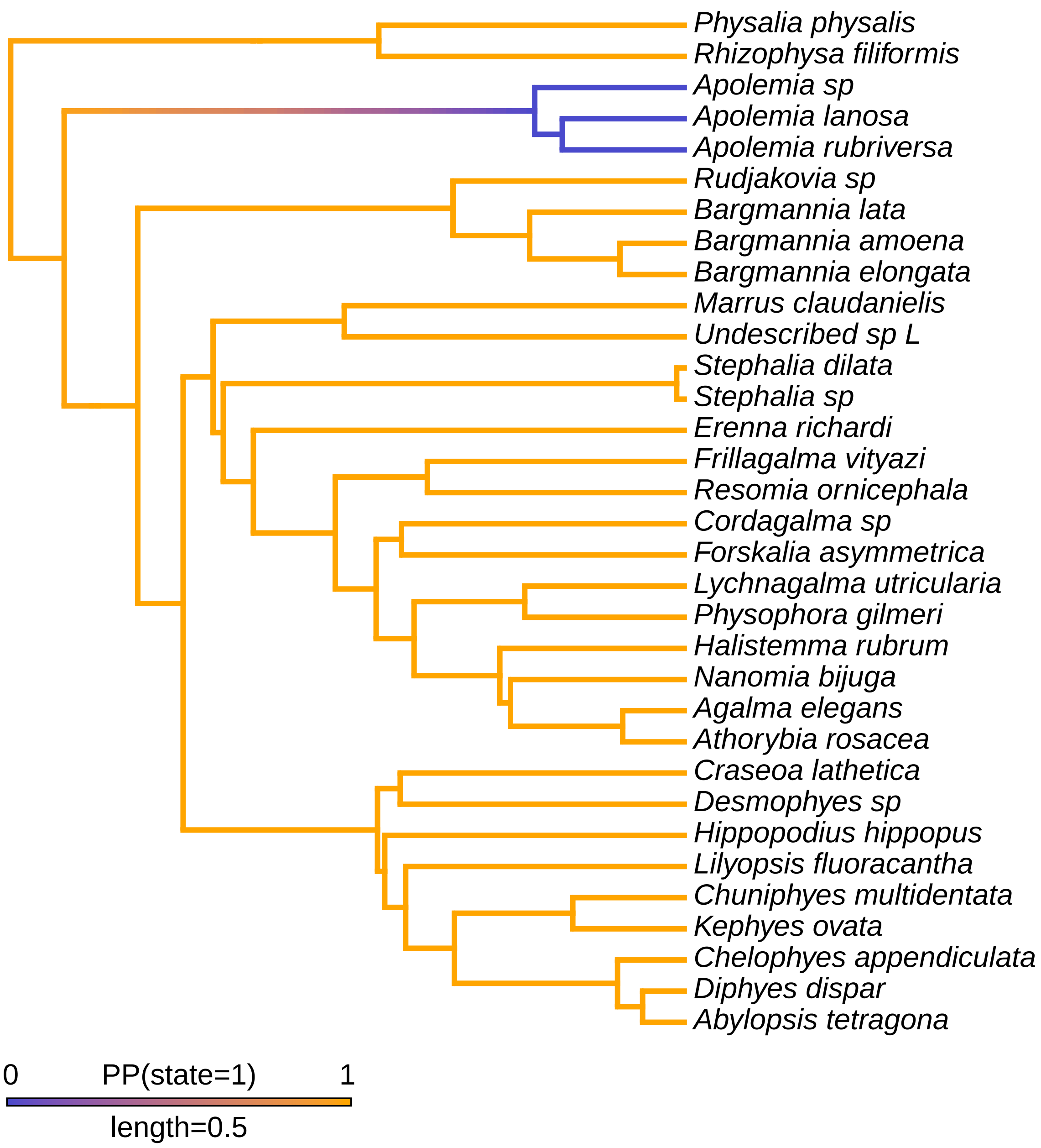

Figure S16: Stochastic character map of presence of tentilla with *Physalia* included as bearing tentilla, mapped on the consensus Bayesian tree topology.

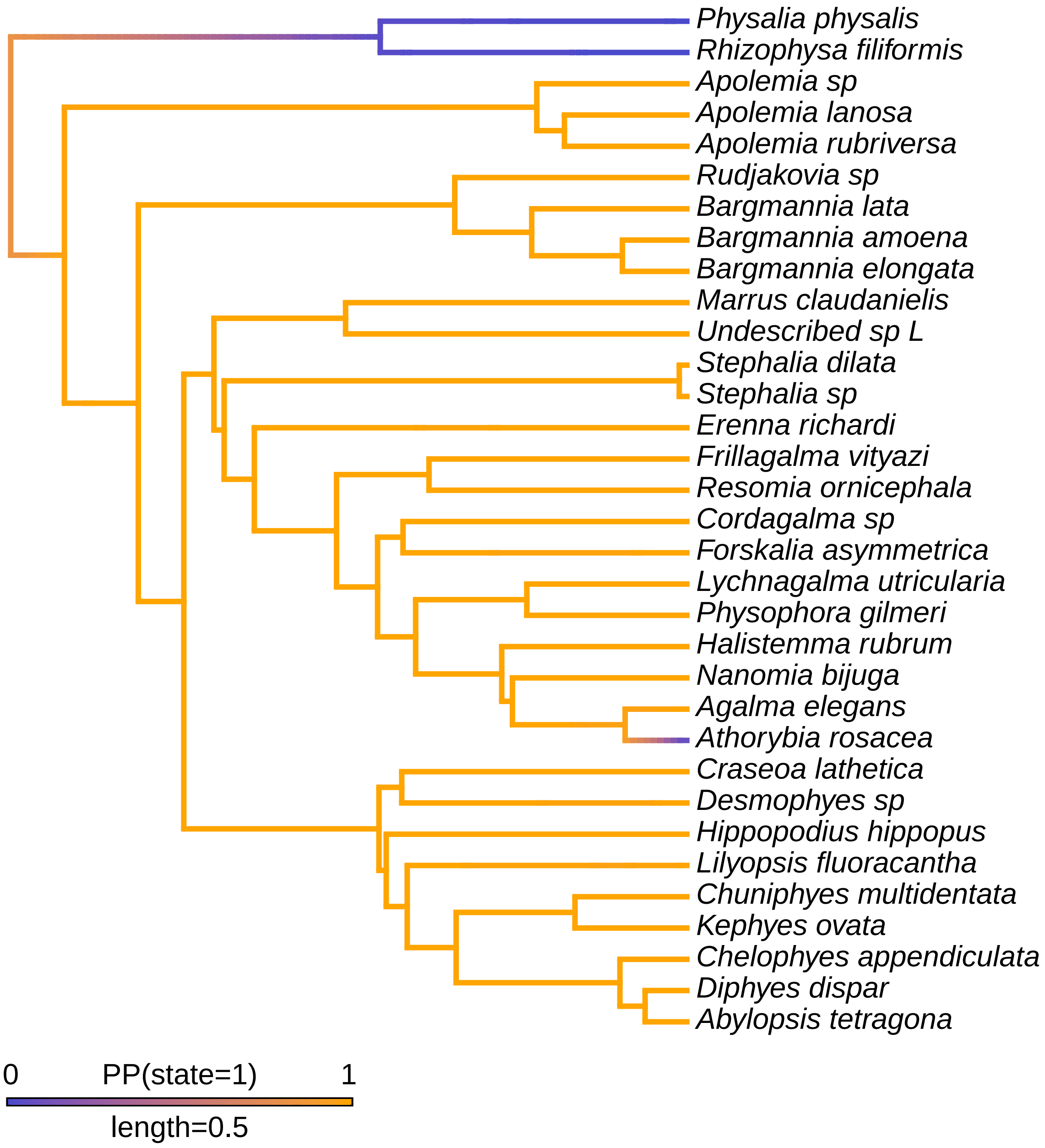

Figure S17: Stochastic character map of presence of nectosome, mapped on the consensus Bayesian tree topology.

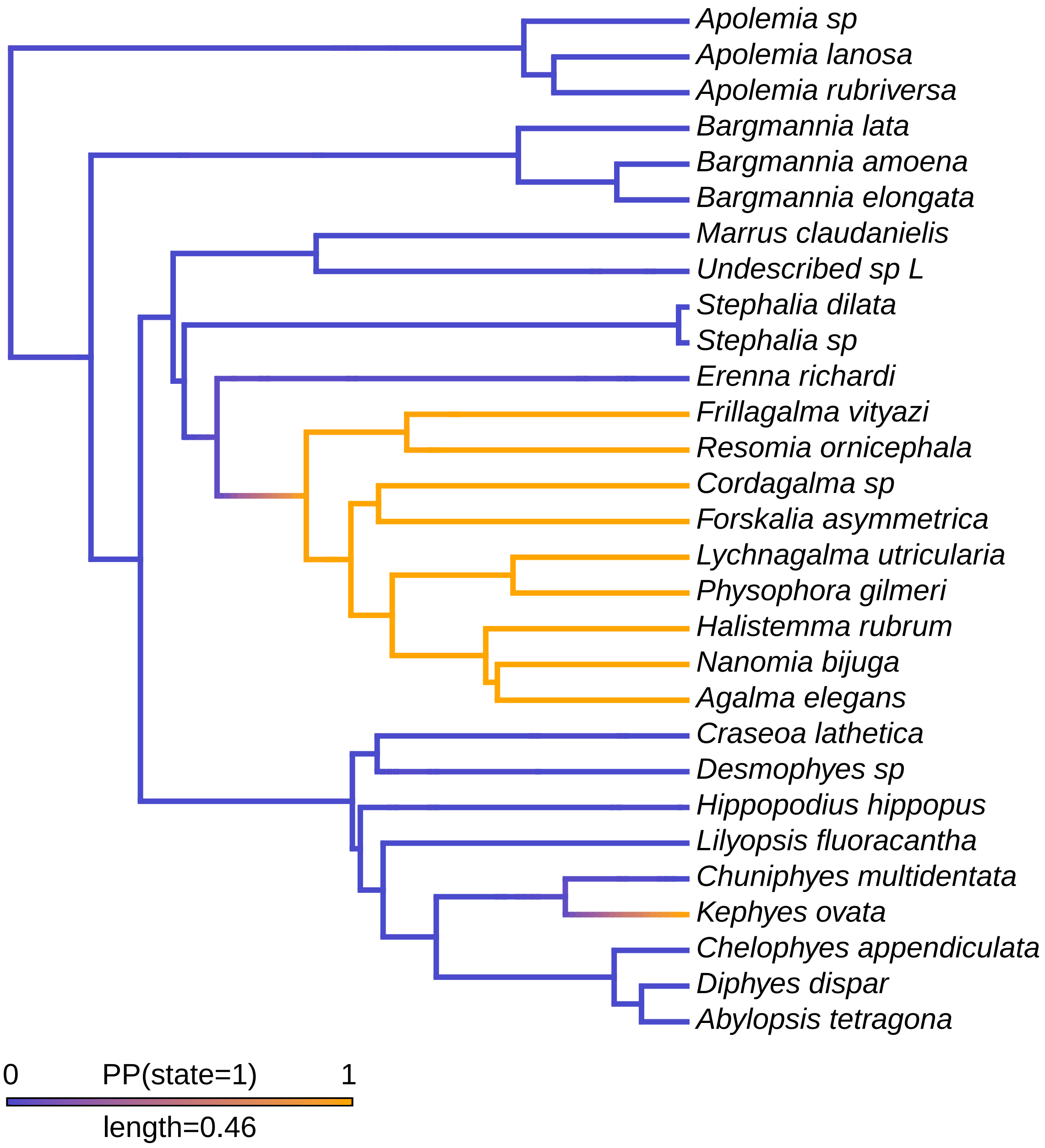

Figure S18: Stochastic character map of presence of a descending mantle canal in the nectophores, mapped on the consensus Bayesian tree topology.

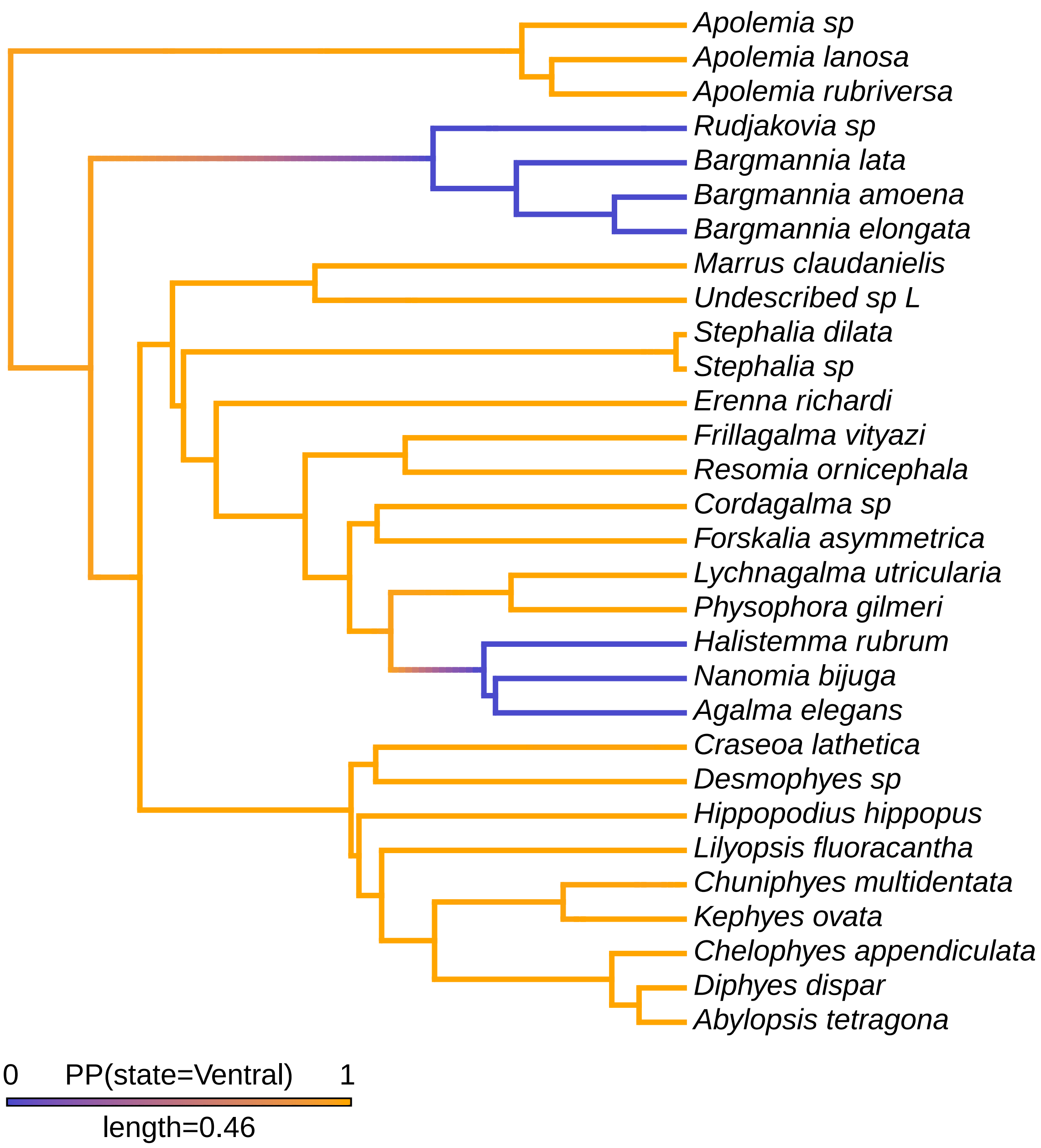

Figure S19: Stochastic character map for the evolution of the position of the nectosome, mapped on the consensus Bayesian tree topology.

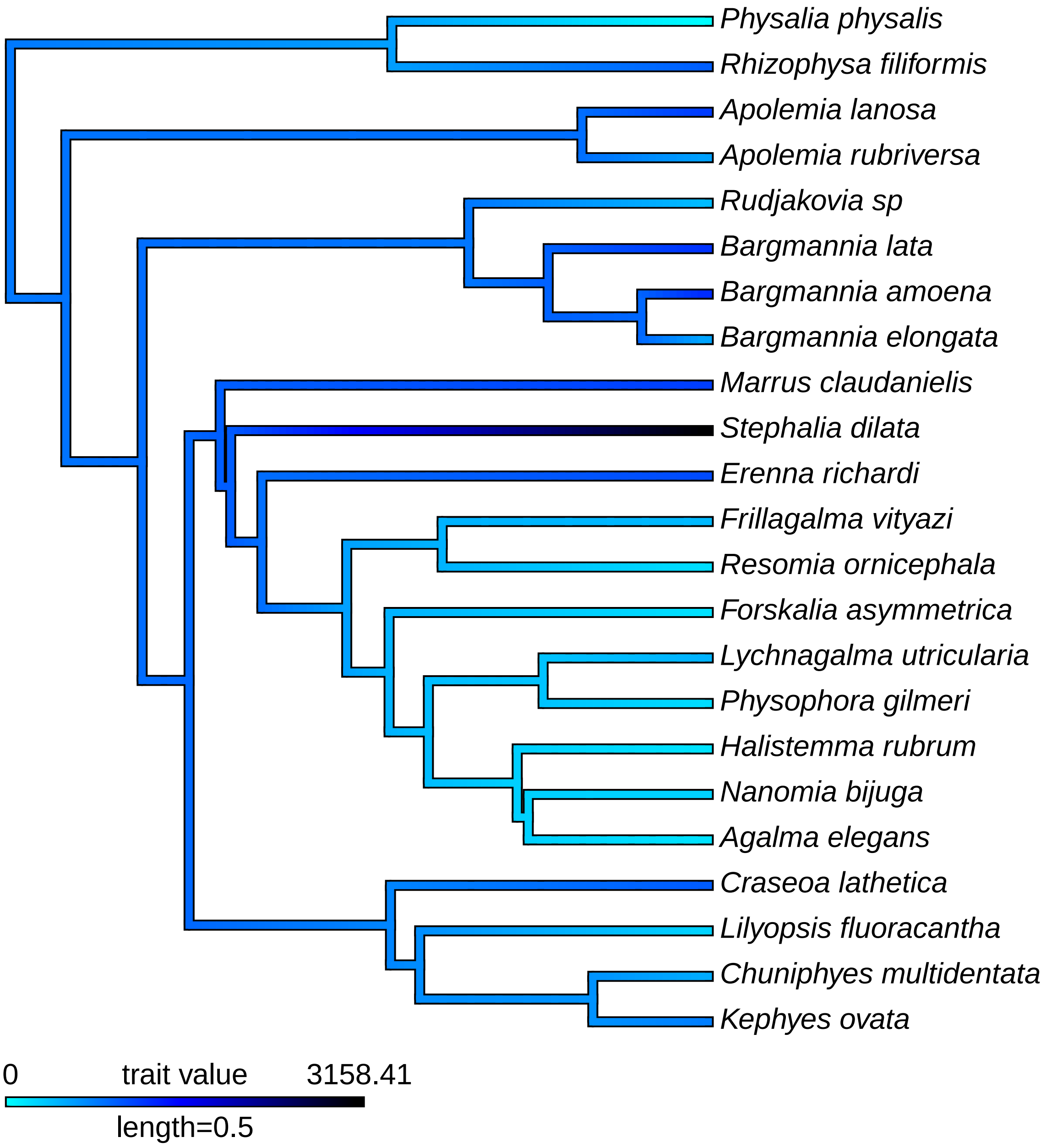

Figure S20: Brownian Motion character map of median depth of species observed with an MBARI ROV, mapped on the consensus Bayesian tree topology.

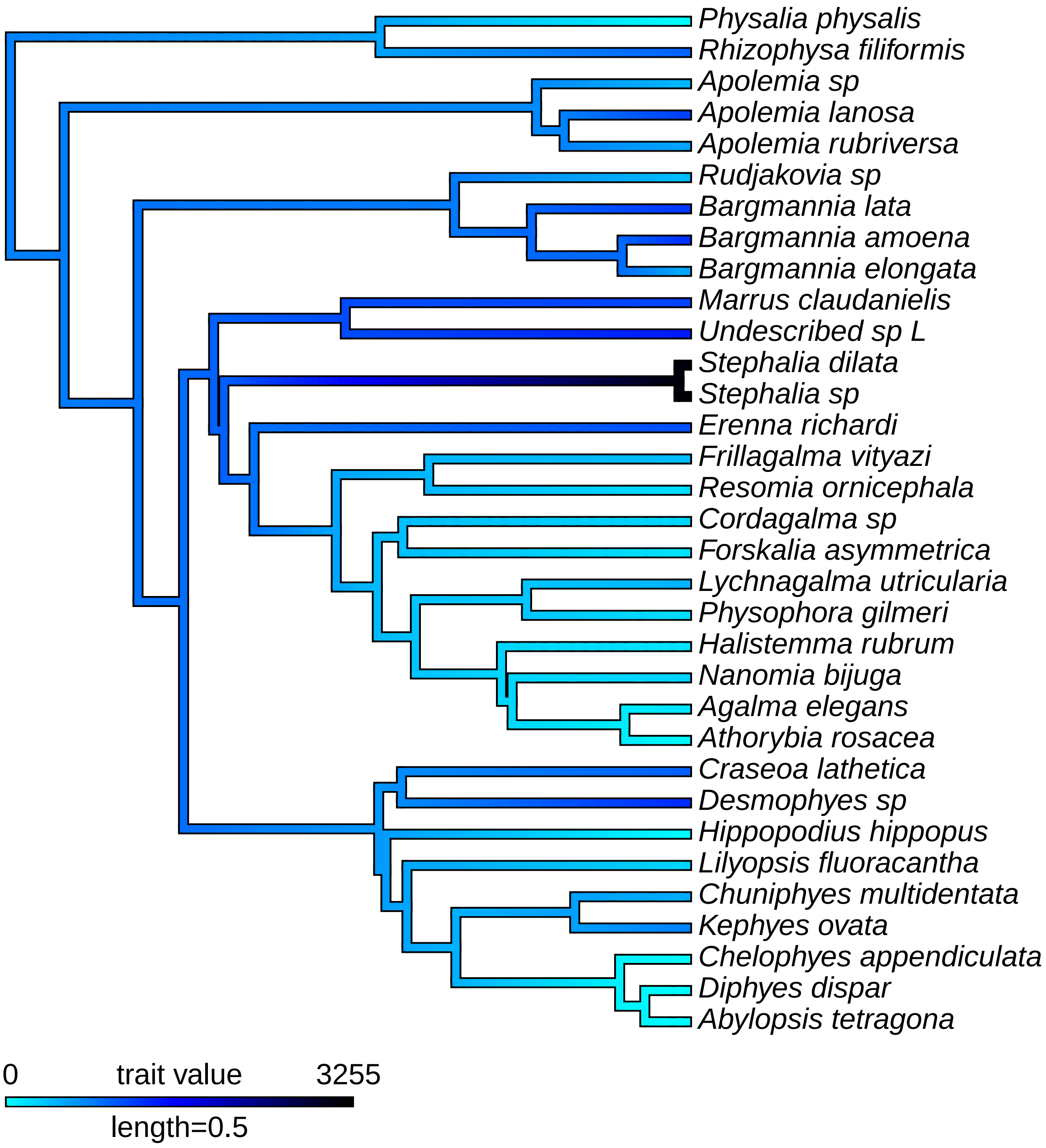

Figure S21: Brownian Motion character map of median depth of species including blue water diving observations, mapped on the consensus Bayesian tree topology.

Software versions

This manuscript was computed on Fri May 18 17:24:11 2018 with the following R package versions.

```
R version 3.4.3 (2017-11-30)
Platform: x86_64-apple-darwin15.6.0 (64-bit)
Running under: macOS High Sierra 10.13.4

Matrix products: default
BLAS: /Library/Frameworks/R.framework/Versions/3.4/Resources/lib/libRblas.0.dylib
LAPACK: /Library/Frameworks/R.framework/Versions/3.4/Resources/lib/libRlapack.dylib

locale:
[1] en_GB.UTF-8/en_GB.UTF-8/en_GB.UTF-8/C/en_GB.UTF-8/en_GB.UTF-8

attached base packages:
[1] grid      parallel  stats     graphics  grDevices utils     datasets
[8] methods   base

other attached packages:
 [1] bindrcpp_0.2.2    phylolm_2.5       geomorph_3.0.6
 [4] rgl_0.99.16       adephylo_1.1-11   ade4_1.7-11
 [7] phylobase_0.8.4   geiger_2.0.6      phangorn_2.4.0
[10] phytools_0.6-44   picante_1.6-2     nlme_3.1-137
[13] vegan_2.5-1       lattice_0.20-35   permute_0.9-4
[16] ape_5.1           hutan_0.5.1       FactoMineR_1.40
[19] factoextra_1.0.5  gridExtra_2.3     seriation_1.2-3
[22] fields_9.6        maps_3.3.0        spam_2.1-4
[25] dotCall64_0.9-5.2 ggtree_1.10.5     treeio_1.2.2
[28] cowplot_0.9.2     xtable_1.8-2      jsonlite_1.5
[31] knitr_1.20        digest_0.6.15     magrittr_1.5
[34] forcats_0.3.0     stringr_1.3.0     dplyr_0.7.4
[37] purrr_0.2.4       readr_1.1.1       tidyr_0.8.0
[40] tibble_1.4.2      ggplot2_2.2.1     tidyverse_1.2.1

loaded via a namespace (and not attached):
  [1] tidyselect_0.2.4        htmlwidgets_1.2
  [3] TSP_1.1-6               combinat_0.0-8
  [5] trimcluster_0.1-2       RNeXML_2.0.8
  [7] munsell_0.4.3           animation_2.5
  [9] codetools_0.2-15        miniUI_0.1.1
 [11] colorspace_1.3-2        highr_0.6
 [13] uuid_0.1-2              rstudioapi_0.7
 [15] leaps_3.0               stats4_3.4.3
 [17] robustbase_0.92-8       labeling_0.3
 [19] mnormt_1.5-5            rprojroot_1.3-2
 [21] coda_0.19-1             LearnBayes_2.15.1
 [23] clusterGeneration_1.3.4 xfun_0.1
 [25] adegenet_2.1.1          gclus_1.3.1
 [27] diptest_0.75-7          R6_2.2.2
 [29] taxize_0.9.3            manipulateWidget_0.9.0
 [31] flexmix_2.3-14          bitops_1.0-6
 [33] reshape_0.8.7           assertthat_0.2.0
 [35] promises_1.0.1          scales_0.5.0
```

```
 [37] nnet_7.3-12            gtable_0.2.0
 [39] bold_0.5.0             rlang_0.2.0
 [41] scatterplot3d_0.3-41   splines_3.4.3
 [43] lazyeval_0.2.1         broom_0.4.4
 [45] yaml_2.1.19            reshape2_1.4.3
 [47] modelr_0.1.1           crosstalk_1.0.0
 [49] backports_1.1.2        httpuv_1.4.1
 [51] tools_3.4.3            bookdown_0.7
 [53] psych_1.8.3.3          spData_0.2.8.3
 [55] gplots_3.0.1           Rcpp_0.12.16
 [57] plyr_1.8.4             progress_1.1.2
 [59] prettyunits_1.0.2      deldir_0.1-15
 [61] viridis_0.5.1          deSolve_1.20
 [63] zoo_1.8-1              haven_1.1.1
 [65] ggrepel_0.7.0          cluster_2.0.7-1
 [67] crul_0.5.2             data.table_1.10.4-3
 [69] gmodels_2.16.2         mvtnorm_1.0-7
 [71] whisker_0.3-2          hms_0.4.2
 [73] mime_0.5               evaluate_0.10.1
 [75] XML_3.98-1.11          jpeg_0.1-8
 [77] mclust_5.4             readxl_1.1.0
 [79] compiler_3.4.3         KernSmooth_2.23-15
 [81] crayon_1.3.4           htmltools_0.3.6
 [83] mgcv_1.8-23            later_0.7.2
 [85] spdep_0.7-7            expm_0.999-2
 [87] lubridate_1.7.4        DBI_1.0.0
 [89] subplex_1.5-4          MASS_7.3-50
 [91] fpc_2.1-11             boot_1.3-20
 [93] Matrix_1.2-14          cli_1.0.0
 [95] quadprog_1.5-5         gdata_2.18.0
 [97] bindr_0.1.1            igraph_1.2.1
 [99] pkgconfig_2.0.1        flashClust_1.01-2
[101] rncl_0.8.2             rvcheck_0.0.9
[103] registry_0.5           numDeriv_2016.8-1
[105] foreign_0.8-70         sp_1.2-7
[107] xml2_1.2.0             foreach_1.4.4
[109] rvest_0.3.2            msm_1.6.6
[111] rmarkdown_1.9          cellranger_1.1.0
[113] fastmatch_1.1-0        dendextend_1.8.0
[115] curl_3.2               kernlab_0.9-25
[117] shiny_1.0.5            gtools_3.5.0
[119] modeltools_0.2-21      seqinr_3.4-5
[121] viridisLite_0.3.0      pillar_1.2.2
[123] httr_1.3.1             plotrix_3.7
[125] DEoptimR_1.0-8         survival_2.42-3
[127] glue_1.2.0             prabclus_2.2-6
[129] iterators_1.0.9        class_7.3-14
[131] stringi_1.2.2          caTools_1.17.1
```
